# Supplementary material for: ATRX promotes heterochromatin formation to protect cells from G-quadruplex DNA-mediated stress
Source: Nat Commun. 2021 Jun 23;12:3887. doi: 10.1038/s41467-021-24206-5 (PMC8222256; doi:10.1038/s41467-021-24206-5)
Supplement: Supplementary file 1 — Supplementary Information [file 41467_2021_24206_MOESM1_ESM.pdf]

ATRX promotes heterochromatin formation to protect cells from G-quadruplex DNA-mediated stress

Authors
Yu-Ching Teng[1], Aishwarya Sundaresan[1], Ryan O'Hara[1], Vincent U. Gant[1], Minhua Li[1], Sara Martire[1], Jane N. Warshaw[1], Amrita Basu[2], Laura A. Banaszynski[1*]

Affiliation
[1]Cecil H. and Ida Green Center for Reproductive Biology Sciences, Department of Obstetrics and Gynecology, Children's Medical Center Research Institute, Harold. C. Simmons Comprehensive Cancer Center, Hamon Center for Regenerative Science and Medicine, University of Texas Southwestern Medical Center, Dallas, Texas 75390, USA.

[2]Department of Surgery, University of California San Francisco, 550 16th St. San Francisco, California 94158, USA

*Correspondence: Laura.Banaszynski@UTSouthwetsern.edu

Table of Contents

Supplementary Methods

Supplementary Figures

Supplementary Figure 1. ATRX is enriched at repetitive elements and predicted G4 elements.
Supplementary Figure 2. Cell cycle analysis of synchronized ESCs.
Supplementary Figure 3. Negative controls for proximity ligation assay.
Supplementary Figure 4. Validation of interaction between ATRX and MCM proteins.
Supplementary Figure 5. ATRX-Mcm6 interaction does not require DAXX.
Supplementary Figure 6. ATRX-Mcm6 interaction is partially reduced in H3.3 KO.
Supplementary Figure 7. ATRX requires its helicase and chaperone activity for preventing G4 formation at sites of DNA synthesis.
Supplementary Figure 8. G4 prediction on observed G4 peaks of CUT&Tag.
Supplementary Figure 9. Analysis of G4-related DNA synthesis and origin activity.
Supplementary Figure 10. DAXX KO and H3.3 KO cells are sensitive to the G4 stabilizer, PDS.
Supplementary Figure 11. ATRX/DAXX complex does not contribute uniformly to transcriptional activity of genes containing ATRX-enriched G4 regions.
Supplementary Figure 12. ESET-mediated heterochromatin formation at ATRX-enriched G4 regions.
Supplementary Figure 13. Mutations at G4 regions are not correlated with *KRAS* or *IDH1* mutations in human tumors.

Supplementary Tables

Supplementary Table 1. List of published genomic data sets analyzed in this study.
Supplementary Table 2. List of antibodies used in this study.
Supplementary Table 3. List of primers used in H3K9me3 ChIP.

Supplementary References

Supplementary Methods

Antibodies. All antibodies used in this study are listed in Supplementary Table 2.

Immunoblot. Cell lysates were generated using digestion buffer (50 mM Tris-HCl, pH 7.6, 1 mM $CaCl_2$, 0.2% Triton X-100 and protease inhibitor cocktail (Roche)) with micrococcal nuclease for 5min at 37°C and denatured in SDS loading buffer. 1.5 x 10^4 cell lysates were run on NuPAGE 4-12% Bis-Tris gel at 180V. Then, the protein on the gel was transferred onto a PVDF membrane (Millipore). The membrane was incubated with antibodies in 5% milk/TBST (0.1% Tween 20) overnight at 4°C, washed, incubated with secondary antibody and developed on the ChemiDoc MP camera system (Bio-Rad). Blot is representative of three independent experiments.

Cell cycle profiling in ESCs. Cell cycle phase analysis was performed using the Click-iT EdU Alexa Fluor 488 Flow Cytometry Assay kit (Thermo Fisher, C10425) according to the manufacturer's instructions. ESCs were grown overnight in a plate. Cells were incubated in medium with 2 mM thymidine for 14h, washed, and incubated in medium with 50 ngml^{-1} nocodazole for 7h[1]. After wash, mitotic cells were labeled with 10 μM EdU for 30 min prior to fixation. 1x 10^6 cells were trypsinized, washed with 1% BSA/PBS, fixed with Click-it fixative, permeabilized with Click-it saponin-based permeabilization and wash reagent and incubated with Click-it reaction (see kit manual). Cells were washed and resuspended in Click-it saponin-based permeabilization and wash reagent with 2 drops of SYTOX AADvanced™ Ready Flow Reagent™ (Thermo Fisher, R37173). Flow cytometry performed on a BD FACSCanto™ II (BD Biosciences). SYTOX AADvanced dye fluorescence was excited by 488 nm laser light and detected in the far red range of the spectrum. Alexa Fluor 488 fluorescence was excited by 488 nm laser light and detected in the green range of the spectrum. Cell cycle analysis was performed using FlowJo software.

Proximity ligation assay (PLA). Cells were seeded on 8 μgml^{-1} fibronectin-coated round cover glass on a 12-well plate. Cell cycle synchronization was performed by incubating cells with 2 mM thymidine for 14h and following 50 ngml^{-1} nocodazole treatment for 7h[1]. Mitotic cells were released and incubated with 2 μM EdU for 20min in prior to time point. Cells were fixed with 4% paraformaldehyde in PBS pH 7.4 for 10min at room temperature then permeabilized in 0.5% Triton X-100 in PBS for 15min.

For ATRX and MCM foci, cells were fixed and permeabilized as described previously. Click-iT reaction and 488-azide were used for detection of EdU-labeling cells. PLA was the same as described previously but using ATRX (ab97508, 1:2500) and Mcm (1:2500) antibodies.

Chromatin immunoprecipitation. Crosslinking ChIP was performed according to published methods[2,3]. Cells were fixed with 2 mM EGS (Thermo Fisher, 21565) for 45min at room temperature, 1% paraformaldehyde for 10min and quenched with 0.125 M glycine. Lysate was sonicated in lysis buffer (1% SDS, 10 mM EDTA, 50 mM Tris, pH 8 and protease inhibitor cocktail) to get 150-500 bp chromatin using a Covaris M220 Focused-ultrasonicator and were immunoprecipitated with ATRX antibody or GFP antibody bound to Dynabeads Protein G overnight at 4°C, with 5% kept as input DNA. Magnetic beads were washed, chromatin was eluted and ChIP DNA was purified by Qiagen PCR purification column. Native ChIP was

performed according to published methods[4]. Cells were collected, washed, and lysed in digestion buffer (50 mM Tris, pH 7.4, 1mM $CaCl_2$, 0.2% Triton X-100, and protease inhibitor cocktail) with micrococcal nuclease for 5min at 37°C. Nuclei were sonicated briefly and dialyzed into RIPA buffer (10 mM Tris, pH 7.6, 1 mM EDTA, 0.1% SDS, 0.1% Na-deoxycholate, 1% Triton X-100) for 2h at 4°C. Soluble materials containing mono- to tri-nucleosomes were incubated with H3K9me3 antibody bound to Dynabeads Protein G overnight at 4°C, with 5% kept as input DNA. Magnetic beads were washed, chromatin was eluted and ChIP DNA was purified by Qiagen PCR purification column. The Spike-in chromatin (Active Motif, 53083) and Spike-in antibody were used in all ChIP experiments according to the manufacturer's instructions.

ChIP-qPCR. qPCR was performed in triplicate using a LightCycler 480 Instrument II system and Power SYBR Green PCR master mix. ChIP DNA samples were diluted 1:100 in water, with 5 µl used per reaction. ChIP–qPCR signal is represented as percent input. All qPCR primer sequences used in this study are listed in Supplementary Table 3.

ATAC-seq. ATAC-seq was performed according to a published protocol[5]. Cells were collected, washed and lysed in lysis buffer (10 mM Tris, pH 7.4, 10 mM NaCl, 3 mM $MgCl_2$, and 0.1%NP-40). Nuclei were collected and subjected to transposase reaction cocktail (25 µl 2X TD buffer, 2.5 µl transposase (Illumina) and 22.5 µl nuclease-free water) at 37°C for 30min. DNA was immediately collected using Qiagen MinElute kit and eluted in 10 mM Tris, pH 8. Eluted DNA was amplified using a KAPA non-hot-start PCR kit with Nextera PCR primer 1 and 2. Libraries were amplified no more than 11 cycles and purified using AMPure XP beads. The quality of libraries was determined using a D5000 ScreenTape on a 2200 TapeStation and a Qubit dsDNA HS Assay kit. Libraries were paired-end 33-base sequenced on the Illumina NextSeq 500. Typical sequencing depth was at least 40 million reads per sample.

Analysis of ATAC-seq data. Quality of the ATAC-seq data sets was assessed using the FastQC tool (v.0.11.2). The ATAC-seq reads were then aligned to the mouse reference genome (mm10) using BWA (v.0.7.5). For unique alignments, duplicate reads were filtered out. The resulting uniquely mapped reads were normalized to the same read depth across all samples and converted into bigWig files using BEDTools (v.2.29.0) and then converted to bigwig using bedGraphToBigWig utility of UCSC kent tools (v317) for visualization in Integrative Genomics Viewer (v.2.3).

CUT&Tag. CUT&Tag was performed according to published methods[6,7]. Cells were collected, washed and lysed in nuclear extraction buffer (20 mM HEPES-KOH, pH 7.9, 10 mM KCl, 0.1% Triton X-100, 20% glycerol, 0.5 mM spermidine, and proteinase inhibitor cocktail) on ice for 10min. While cells were lysed, concanavalin A coated magnetic beads (Epicypher, 21-1401) were activated in binding buffer (20 mM HEPES-KOH, pH 7.9, 10 mM KCl, 1 mM $CaCl_2$, and 1 mM $MnCl_2$). 5 x 10^5 nuclei per sample were washed with PBS once, resuspended in 100 µl wash buffer (20 mM HEPES-KOH, pH 7.5, 150 mM NaCl, 0.5 mM spermidine, and proteinase inhibitor cocktail) and incubated with 10 µl activated beads for 10min at room temperature. Nuclei-bound beads were resuspended in 50 µl antibody buffer (2 mM EDTA and 0.1% BSA in wash buffer) containing 2 µl BG4 antibody and incubated at 4°C overnight. Unbound antibodies were removed and beads were resuspended in 50 µl antibody buffer containing 1 µl anti-DYKDDDK antibody and incubated at room temperature for 2h. After removing unbound antibodies, beads were resuspended in 100 µl wash buffer containing 1 µl anti-rabbit secondary

antibody and incubated at room temperature for 30min. Beads were washed in 1 ml wash buffer three times, resuspended in 100 µl wash-300 buffer (20 mM HEPES-KOH, pH 7.5, 300 mM NaCl, 0.5 mM spermidine, and proteinase inhibitor cocktail) containing 2.5 µl pAG-Tn5 (Epicypher, 15-1017) and incubated at room temperature for 1h. Beads were washed in 1 ml wash-300 buffer three times to remove unbound pAG-Tn5. Next, beads were resuspended in 300 µl tagmentation buffer (10 mM $MgCl_2$ in wash-300 buffer) and incubated at 37°C for 1h. To stop tagmentation, 10 µl of 0.5 M EDTA, 3 µl of 10% SDS, and 2.5 µl of Proteinase K were added to 300 µl of sample, which was incubated at 55°C for 1h. To extract DNA, samples were transferred to Phase Lock Gel tube (QuantaBio, 2302820) and equal volume of Phenol:Chloroform:Isoamyl Alcohol was added into the tube and spin at 13000 rpm for 5min. Supernatant containing DNA was precipitated in 2.5 volumes of absolute alcohol and incubated at -20°C for 30min to overnight. DNA was pelleted after centrifuge and dissolved in 25 µl 0.1x TE. To amplify libraries, DNA was amplified using a KAPA non-hot-start PCR kit with Nextera PCR primer 1 and 2. Libraries were amplified for 12 cycles and purified using AMPure XP beads. The quality of libraries was determined using a D5000 ScreenTape on a 2200 TapeStation and a Qubit dsDNA HS Assay kit. Libraries were paired-end 33-base sequenced on the Illumina NextSeq 500. Typical sequencing depth was at least 100 million reads per sample.

Analysis of CUT&Tag data. CUT&Tag was analyzed following the methods available at https://yezhengstat.github.io/CUTTag_tutorial/ . Briefly, quality of CUT&Tag data sets was assessed using the FACTQC tool (v.0.11.2). CUT&Tag raw reads were trimmed using TrimGalore(v.0.6.4). The trimmed reads were aligned to the mouse reference genome (mm10) using BOWTIE2 (v.2.3.2). The optical duplicate reads were filtered using the MarkDuplicates tool of Picard (v.2.10.3). The resulting uniquely mapped reads were normalized to the same read depth across all samples. Reads were converted into bedgraph files using BEDTools (v.2.29.0) and the. Converted to bigwig using bedGraphToBigWig utility of UCSC kent tools (v317) for visualization in Integrative Genomics Viewer (v.2.3).

EdU-seq. EdU-seq was performed according to a published protocol[8]. Mitotic ESCs were released in medium and incubated in medium with 10 µM EdU for 30min before the cells were collected. At the indicated time, cells were washed and immediately fixed with cold 90% methanol overnight at -20°C. Cells were permeabilized with 0.2% Triton X-100 in PBS for 30min at room temperature. After washing, EdU was coupled to a cleavable biotin-azide (Azide-SS-biotin, BroadPharm, BP-22877) using the Click-iT reaction cocktail (Thermo Fisher, C10269). The DNA was purified by phenol/chloroform extraction in Phase Lock Gel, Light, 1.5-ml tube and ethanol precipitation for EdU-labeled DNA isolation. Total 15 µg DNA was sonicated to a size range of 150-500 bp with a Covaris M220 Focused-ultrasonicator. EdU-labeled DNA fragments were pulled down by Dynabeads MyOne streptavidin C1 (Invitrogen) for 15min at room temperature, washed, and eluted in elution solution (10 mM Tris, pH 8) containing fresh 2% β-mercaptoethanol for 1h at room temperature. The eluted DNA was directly used for library preparation. Asynchronized EdU-labeled HeLa DNA fragments were generated as described previously for a spike-in normalization and used in EdU-seq. The EdU-seq libraries were prepared from 5 ng DNA following the Illumina TruSeq protocol. The quality of libraries was determined using a D5000 ScreenTape on a 2200 TapeStation and a Qubit dsDNA HS Assay kit. Libraries were paired-end 33-base sequenced on the Illumina NextSeq 500. Typical sequencing depth was at least 50 million reads per sample.

Analysis of EdU-seq data. Quality of EdU-seq data sets was assessed using the FastQC tool (v.0.11.2). EdU-seq raw reads were aligned separately to the mouse reference genome (mm10) and the spike-in human reference genome (hg19) using BOWTIE2 (v.2.2.8). Only one alignment is reported for each read (either the single best alignment or, if more than one equivalent best alignment was found, one of those matches selected randomly). Duplicate reads were filtered using the MarkDuplicates tool of Picard (v.1.127). The resulting uniquely mapped reads were normalized to the same read depth across all samples. Reads were converted into bedgraph files using BEDTools (v.2.29.0) and then converted to bigwig using bedGraphToBigWig utility of UCSC kent tools (v317) for visualization in Integrative Genomics Viewer (v.2.3).

Analysis of SNS-seq data. Quality of SNS-seq data sets was assessed using the FastQC tool (v.0.11.2). SNS-seq raw reads were aligned to the mouse reference genome (mm10) using BOWTIE2 (v.2.2.8). Only one alignment is reported for each read (either the single best alignment or, if more than one equivalent best alignment was found, one of those matches selected randomly). Duplicate reads were filtered using the MarkDuplicates tool of Picard (v.1.127). The resulting uniquely mapped reads were normalized to the same read depth across all samples. Reads were converted into bedgraph files using BEDTools (v.2.29.0) and then converted to bigwig using bedGraphToBigWig utility of UCSC kent tools (v317) for visualization in Integrative Genomics Viewer (v.2.3).

Analysis of RNA-seq data. Quality of the RNA-seq raw reads was assessed using the FastQC tool (v.0.11.2). The reads were then aligned to the mouse reference genome (mm10) using the spliced read aligner TopHat version v.2.0.12, transcriptome assembly was carried out using Cufflinks v.2.2.1 with default parameters, filtered transcripts were merged into distinct nonoverlapping sets using Cuffmerge, and Cuffdiff was used to calculate the differential expression genes between the conditions.

Teng et al., Supplementary Fig. 1

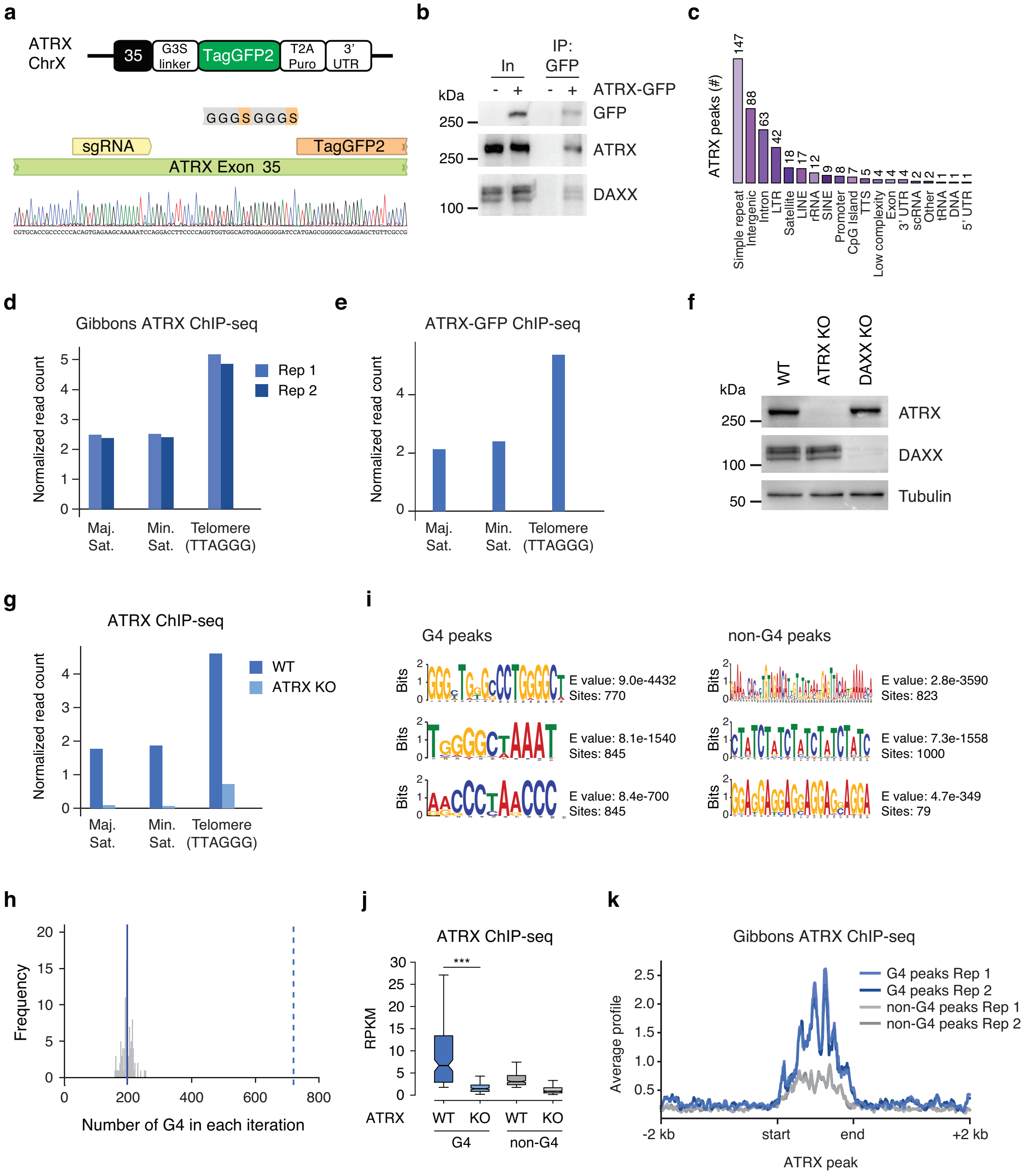

Supplementary Figure 1. ATRX is enriched at repetitive elements and predicted G4 elements. Related to Figure 1. (**a**) Schematic of GFP tagging of the endogenous mouse ATRX gene. An sgRNA targets exon 35 of ATRX gene. A CRISPR/Cas9-mediated homology directed repair system allows selection for positively tagged cells based on expression of a puromycin

resistance gene separated from the GFP sequence by a T2A peptide. Genotyping shows a single-cell clone with the GFP gene inserted in-frame into the ATRX gene. (**b**) Co-immunoprecipitation from ESC nuclear extracts showing ATRX-GFP interaction with DAXX. Data are representative of n>3. (**c**) Peak Annotation. The x axis represents the annotation category and the number above the bar graph represents the number of peaks in the particular annotation category. (**d**) Published ATRX ChIP-seq in ESCs shows ATRX enrichment at telomeres and satellite sequences[2]. (**e**) ATRX-GFP ChIP-seq in ESCs shows ATRX-GFP enrichment at telomeres and satellite sequences. (**f**) Immunoblot from ESC whole-cell lysates showing the expression of ATRX and DAXX in wild-type, ATRX KO and DAXX KO ESCs. Tubulin as loading control. Data are representative of n>3. (**g**) ATRX ChIP-seq in ESCs shows ATRX enrichment at telomeres and satellite sequences. This enrichment is reduced in ATRX KO ESCs. (**h**) The histogram shows the number of a G-quadruplex motif in random sequences compared with the 720 observed number of G-quadruplex motif (dashed line). Simulation was used to estimate the number of G-quadruplex motif by chance. For 100 times, the peaks were randomly shuffled throughout the genome while maintaining the number of peaks and their size and the number of G-quadruplexes computed. On average,198 G-quadruplexes (sd= 17) (solid line) were observed therefore the observed number of G-quadruplex motifs is significantly larger (by 30 standard deviations) than expected by chance. (**i**) Motif enrichment for ATRX-enriched G4 (left) and non-G4 (right) peaks. (**j**) Box plots representing ATRX ChIP-seq in ESCs at ATRX-enriched G4 ($p = 9.1x10^{-35}$) and non-G4 regions in wild-type and ATRX KO cells. The bottom and the top of the boxes correspond to the 25th and 75th percentiles, and the internal band is the 50th percentile (median). The plot whiskers correspond to 1.5 interquartile range. Statistical significance is determined by Wilcoxon Mann Whitney test. ***$P < 0.001$. (**k**) Published ATRX ChIP-seq average profiles in ESCs for ATRX-enriched G4 and non-G4 regions[2]. For all immunoblots, source data are provided as a Source Data file.

Teng et al., Supplementary Fig. 2

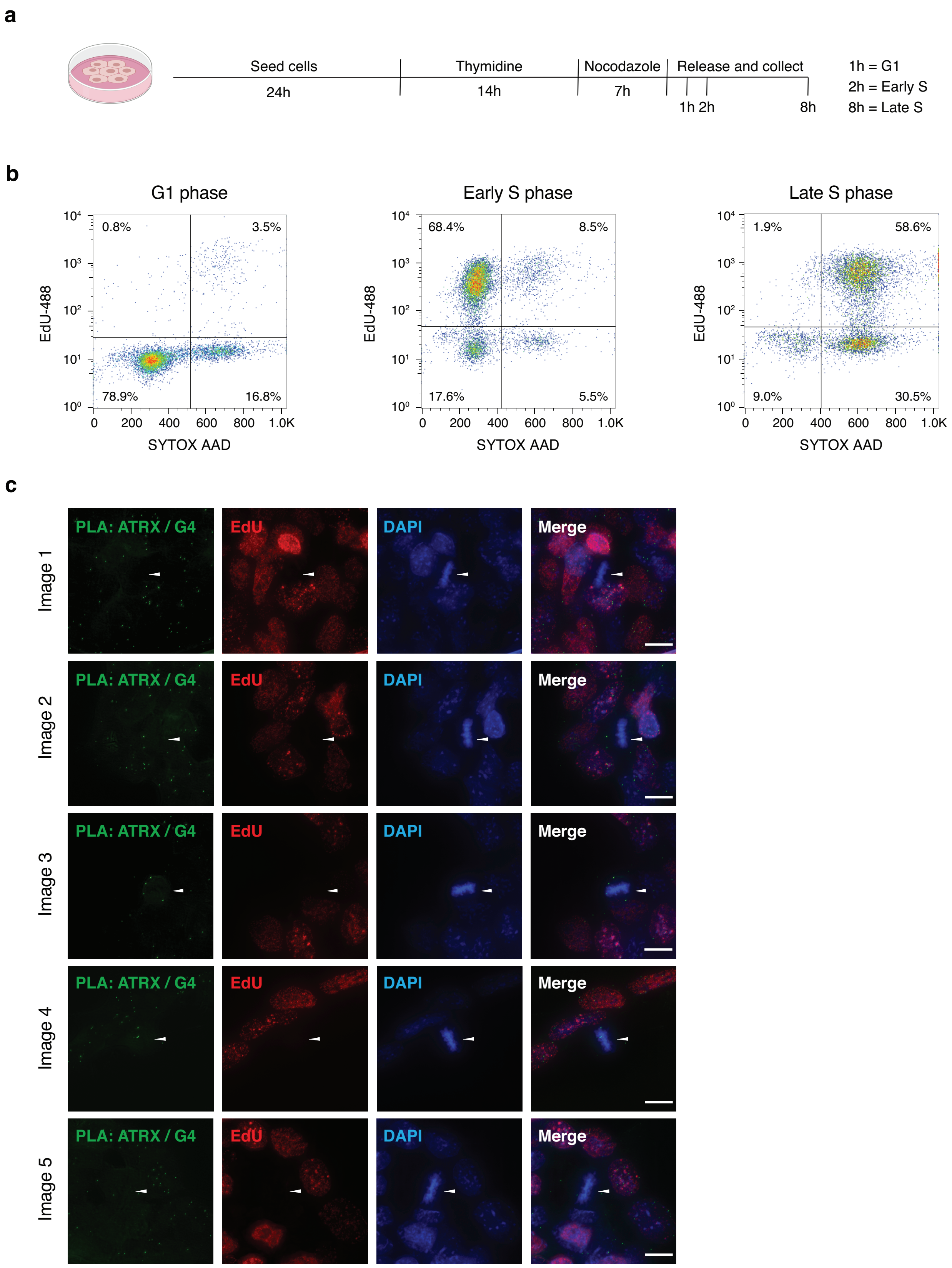

Supplementary Figure 2. Cell cycle analysis of synchronized ESCs. Related to Figure 1. (**a**) Schematic of ESCs synchronization protocol. Cells are incubated with thymidine for 14h, washed, and treated in medium with nocodazole for 7h. After washing, mitotic cells are released in medium and incubated with EdU in prior cell fixation for downstream experiments. Cells in G1, early S, and late S phase were analyzed 1h, 2h, and 8h after release, respectively. (**b**) Cell cycle profiles of ESCs in G1, early S and late S phases. (**c**) Representative images assessing ATRX and G4 colocalization by proximity ligation assay (PLA) in asynchronized ESCs. n = 1 biological replicate. Green - PLA (ATRX-G4). Red - EdU-labeling, indicative of newly synthesized DNA. Blue - DAPI nuclear stain. Scale bar equals 10 μm. Arrows indicate metaphase chromosomes.

Teng et al., Supplementary Fig. 3

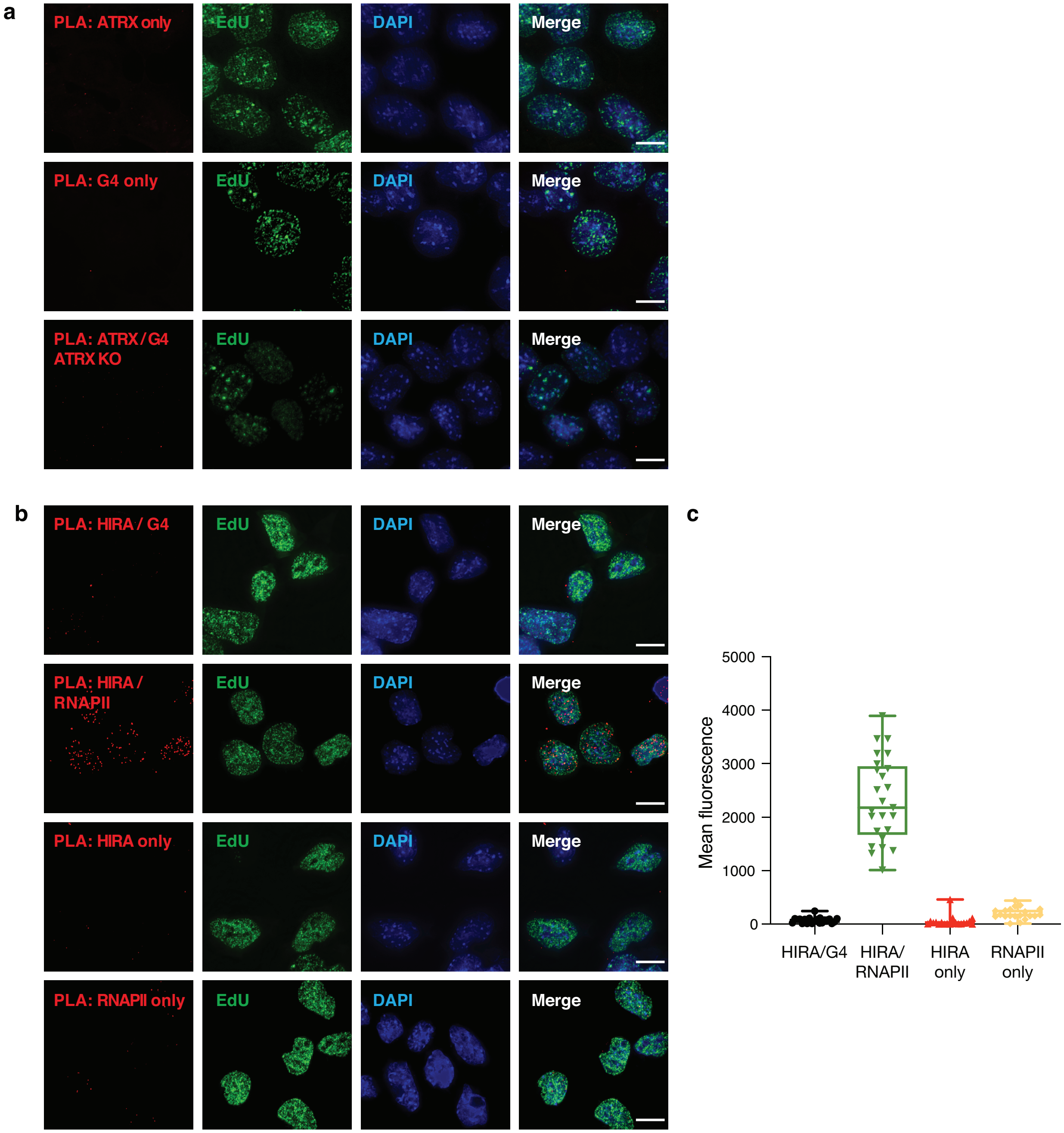

Supplementary Figure 3. Negative controls for proximity ligation assay. Related to Figure 1. (**a**) Representative images displaying PLA foci when ATRX or BG4 antibody alone was applied in ESCs (top and middle panels) and when both ATRX and BG4 antibodies were applied in ATRX KO ESCs (bottom panels). Data are representative of n>3. (**b**) Representative images showing the PLA foci in ESCs when both HIRA and BG4 antibodies were applied (top panels), when both HIRA and RNA polymerase II (RNAPII) antibodies were applied (second panels) and when HIRA or RNAPII antibody alone was applied (third and bottom panels). n=1 biological replicate. For a single antibody experiment, all procedures of PLA were described in methods

except only one antibody used. HIRA and RNAPII antibodies used 1:1,000 dilution. Red - PLA foci. Green - EdU-labeling, indicative of newly synthesized DNA. Blue -DAPI nuclear stain. Scale bar equals 10 µm. (**c**) Quantification of signal intensity from PLA foci in ESCs shown in Supplementary Figure 3b. HIRA/G4 (n=22), HIRA/RNAPII (n=25), HIRA only (n=21), and RNAPII only (n=20). The bottom and the top of the boxes correspond to the 25th and 75th percentiles, and the internal band is the 50th percentile (median). The plot whiskers show down to the minimum and up to the maximum value.

Teng et al., Supplementary Fig. 4

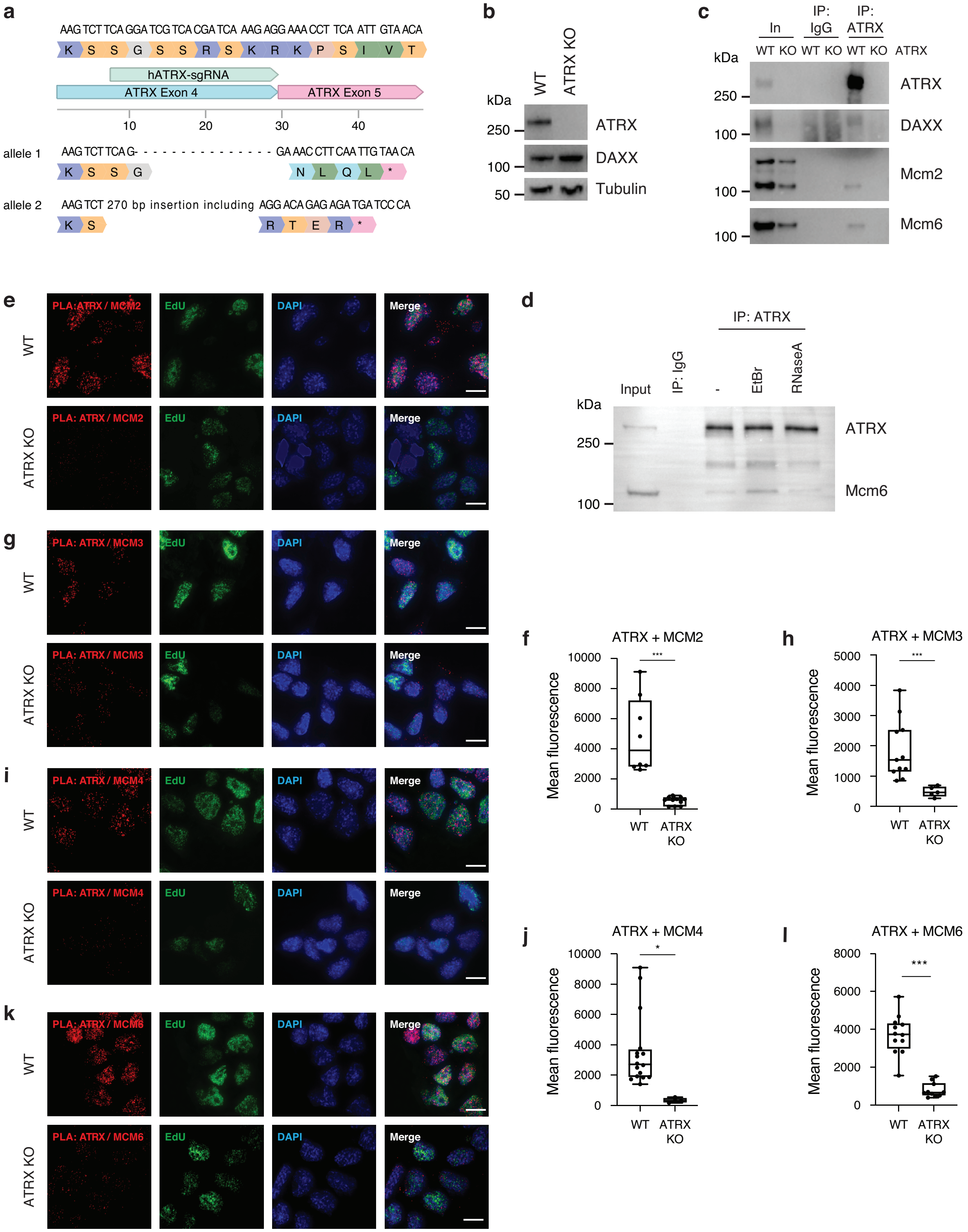

Supplementary Figure 4. Validation of interaction between ATRX and MCM proteins. Related to Figure 2. (**a**) Generation of CRISPR/Cas9 mediated ATRX KO cells. HeLa cells were transfected with a Cas9 expression plasmid containing an sgRNA targeting exon 4 of the ATRX gene. A single clone was isolated and validated by Sanger sequencing. (**b**) Immunoblot from HeLa whole-cell lysates showing the expression of ATRX and DAXX in wild-type and ATRX KO cells. Tubulin as loading control. (**c**) Co-immunoprecipitation from HeLa nuclear extracts showing ATRX interaction with DAXX, MCM2 and MCM6. **(d)** Co-immunoprecipitation from ESC nuclear extracts showing ATRX-Mcm6 interaction in lysates treated with either ethidium bromide or RNase A. Data from panels **b-d** are representative of n>3. (**e, g, i, and k**) Representative images demonstrating ATRX and Mcm2, Mcm3, Mcm4, and Mcm6 co-localization by proximity ligation assay (PLA) in early S phase of wild-type and ATRX KO ESCs, n>3. Red - PLA (ATRX-Mcm proteins). Green - EdU-labeling, indicative of newly synthesized DNA. Blue - DAPI nuclear stain. Scale bar equals 10 μm. (**f, h, j, and l**) Quantification of signal intensity from ATRX-Mcm PLA foci in early S phase of ESCs in Supplementary Figure 4e, 4g, 4i, and 4k. The bottom and the top of the boxes correspond to the 25th and 75th percentiles, and the internal band is the 50th percentile (median). The plot whiskers show down to the minimum and up to the maximum value. Statistical significance is determined by Mann-Whitney U-test. *P < 0.05; ***P < 0.001. In (**f**), wild-type (n=8) and ATRX KO (n=11) ($p = 2.6x10^{-5}$); In (**h**), wild-type (n=11) and ATRX KO (n=5) ($p = 4.6x10^{-4}$); In (**j**), wild-type (n=16) and ATRX KO (n=4) ($p = 4.1x10^{-4}$); In (**l**), wild-type (n=12) and ATRX KO (n=10) ($p = 1x10^{-6}$). For all immunoblots, source data are provided as a Source Data file.

Teng et al., Supplementary Fig. 5

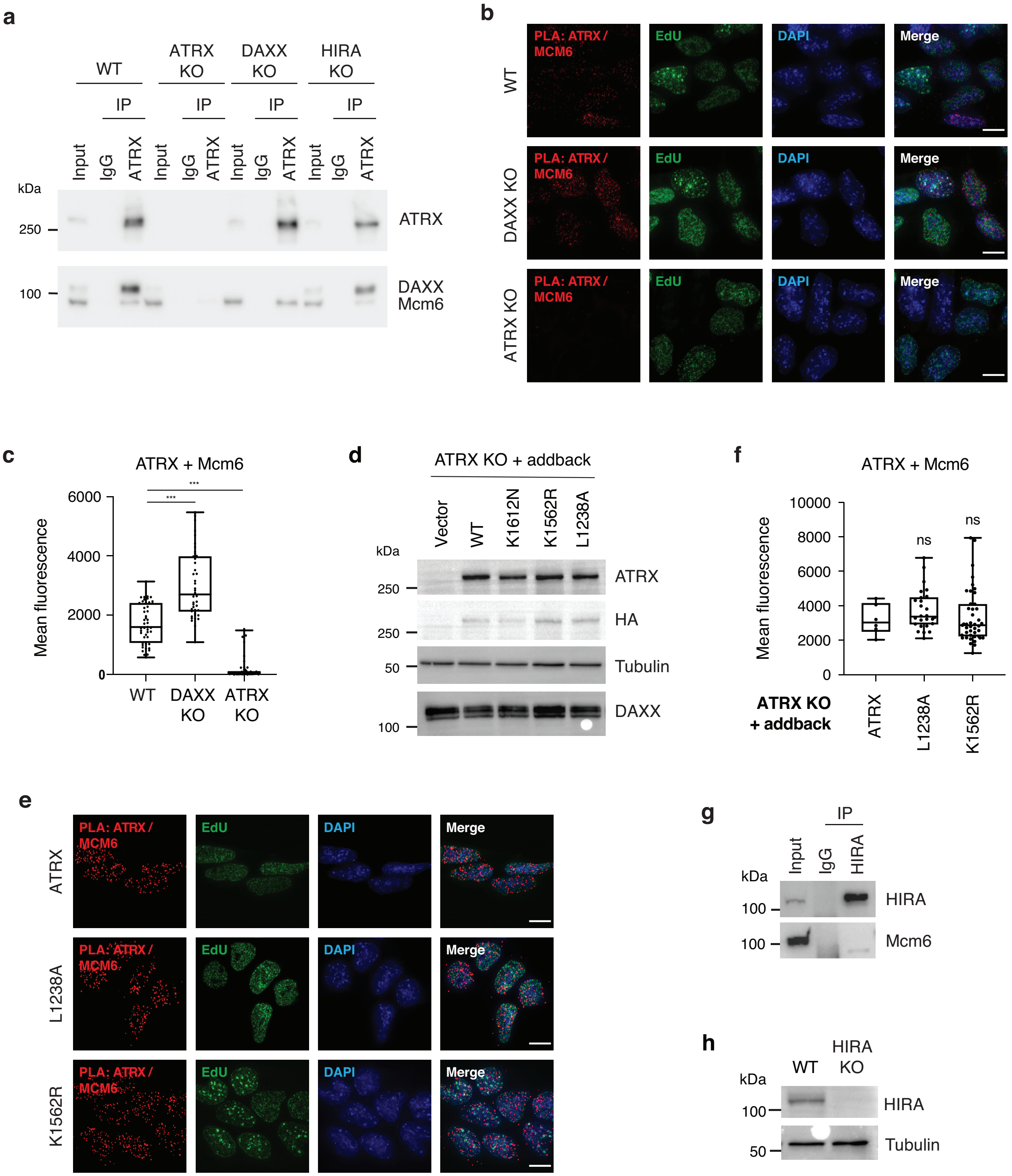

Supplementary Figure 5. ATRX-Mcm6 interaction does not require DAXX. Related to Figure 2. (**a**) Co-immunoprecipitation from ESC nuclear extracts showing ATRX interaction with DAXX and Mcm6 in wild-type, ATRX KO, DAXX KO, and HIRA KO ESCs. n=2 experimental replicates. (**b**) Representative images demonstrating ATRX-Mcm6 co-localization

by proximity ligation assay (PLA) in asynchronized wild-type, ATRX KO (n=47), and DAXX KO ESCs (n=42). (**c**) Quantification of signal intensity from ATRX-Mcm6 PLA foci in Supplementary Figure 5b. The bottom and the top of the boxes correspond to the 25th and 75th percentiles, and the internal band is the 50th percentile (median). The plot whiskers show down to the minimum and up to the maximum value. Statistical significance determined by One-way ANOVA test compared to ATRX KO. WT - $p = 1x10^{-10}$; DAXX KO - $p = 1x10^{-10}$. ***$p < 0.001$. (**d**) Immunoblot from ESC whole-cell lysates showing the expression of wild-type and mutants of HA-tagged ATRX in ATRX KO cells, n>3. DAXX expression remains the same within each cell lysate. (**e**) Representative images demonstrating ATRX-Mcm6 co-localization by proximity ligation assay (PLA) in early S phase of ATRX KO ESCs that expressing exogenous wild-type ATRX and ATRX mutants (L1238A and K1562R). n=2 experimental replicates. (**f**) Quantification of signal intensity from ATRX-Mcm6 PLA foci in Supplementary Figure 5e. ATRX (n=6), L1238A (n=26) ($p = 0.57$), and K1562R (n=43) ($p = 0.80$). The bottom and the top of the boxes correspond to the 25th and 75th percentiles, and the internal band is the 50th percentile (median). The plot whiskers show down to the minimum and up to the maximum value. (**g**) Co-immunoprecipitation from HeLa nuclear extracts showing HIRA does not interact with MCM6. n=1 experimental replicate. (**h**) Immunoblot from ESC whole-cell lysates showing the expression of HIRA in wild-type and HIRA KO cells, n>3. For **d** and **h**, tubulin as loading control. For **b** and **e**, Red - PLA (ATRX-Mcm6). Green - EdU-labeling, indicative of newly synthesized DNA. Blue - DAPI nuclear stain. Scale bar equals 10 μm. Statistical significance determined by One-way ANOVA test. ***$p < 0.001$. ns; not significant. For all immunoblots, source data are provided as a Source Data file.

Teng et al., Supplementary Fig. 6

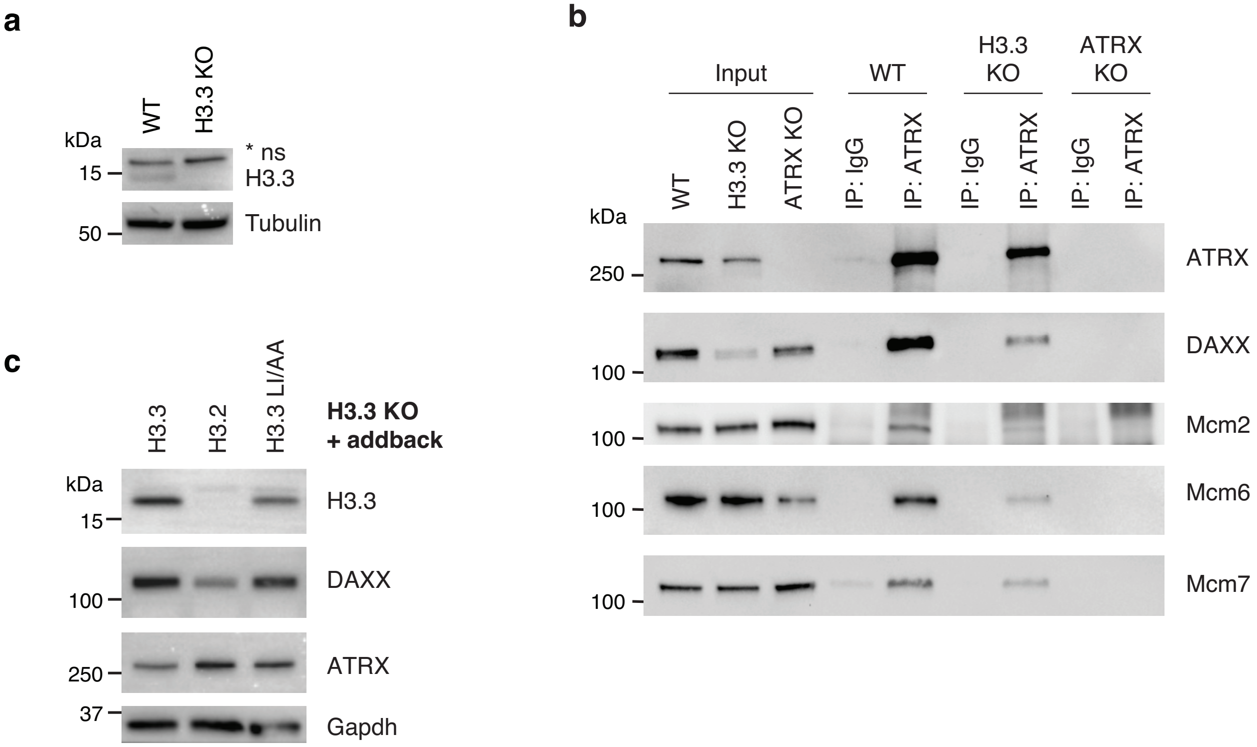

Supplementary Figure 6. ATRX-Mcm6 interaction is partially reduced in H3.3 KO. Related to Figure 2. (**a**) Immunoblot from ESC whole-cell lysates showing H3.3 expression levels in wild-type and H3.3 KO cells, n>3. ns, non-specific band. (**b**) Co-immunoprecipitation from ESC nuclear extracts showing ATRX interaction with DAXX, Mcm2, Mcm6 and Mcm7 in wild-type, H3.3 KO, and ATRX KO cells. n=3 experimental replicates. (**c**) Immunoblot form ESC whole-cell lysates showing the expression of H3.3, DAXX, and ATRX in H3.3 KO cells exogenously expressing H3.3, H3.2 or H3.3 L126A L130A (H3.3 LI/AA), n>3. For **a** and **c**, tubulin and gapdh as loading control. For all immunoblots, source data are provided as a Source Data file.

Teng et al., Supplementary Figure 7

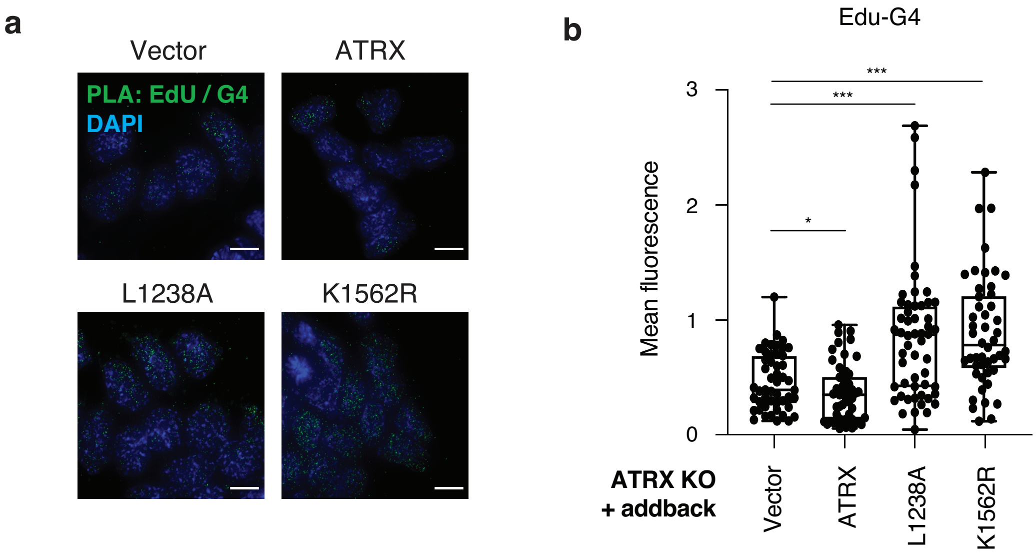

Supplementary Figure 7. ATRX requires its helicase and chaperone activity for preventing G4 formation at sites of DNA synthesis. Related to Figure 3. (**a**) Representative images demonstrating EdU and G4 co-localization by proximity ligation assay (PLA) in early S phase of ATRX KO ESCs exogenously expressing wild-type ATRX (n=53) and ATRX mutants (L1238A, n=54 and K1562R, n=48). n=3 experimental replicates. Green - PLA (EdU-G4). Blue - DAPI nuclear stain. Scale bar equals 10 µm. (**b**) Quantification of signal intensity from EdU-G4 PLA foci in Supplementary Figure 7a. The bottom and the top of the boxes correspond to the 25th and 75th percentiles, and the internal band is the 50th percentile (median). The plot whiskers show down to the minimum and up to the maximum value. Statistical significance determined by a One-way ANOVA test compared to ATRX KO + Vector. ATRX - $p = 0.039$; L1238A - $p = 2.2x10^{-5}$; K1562R - $p = 8.4x10^{-7}$ *$p < 0.05$; ***$p < 0.001$.

Teng et al., Supplementary Figure 8

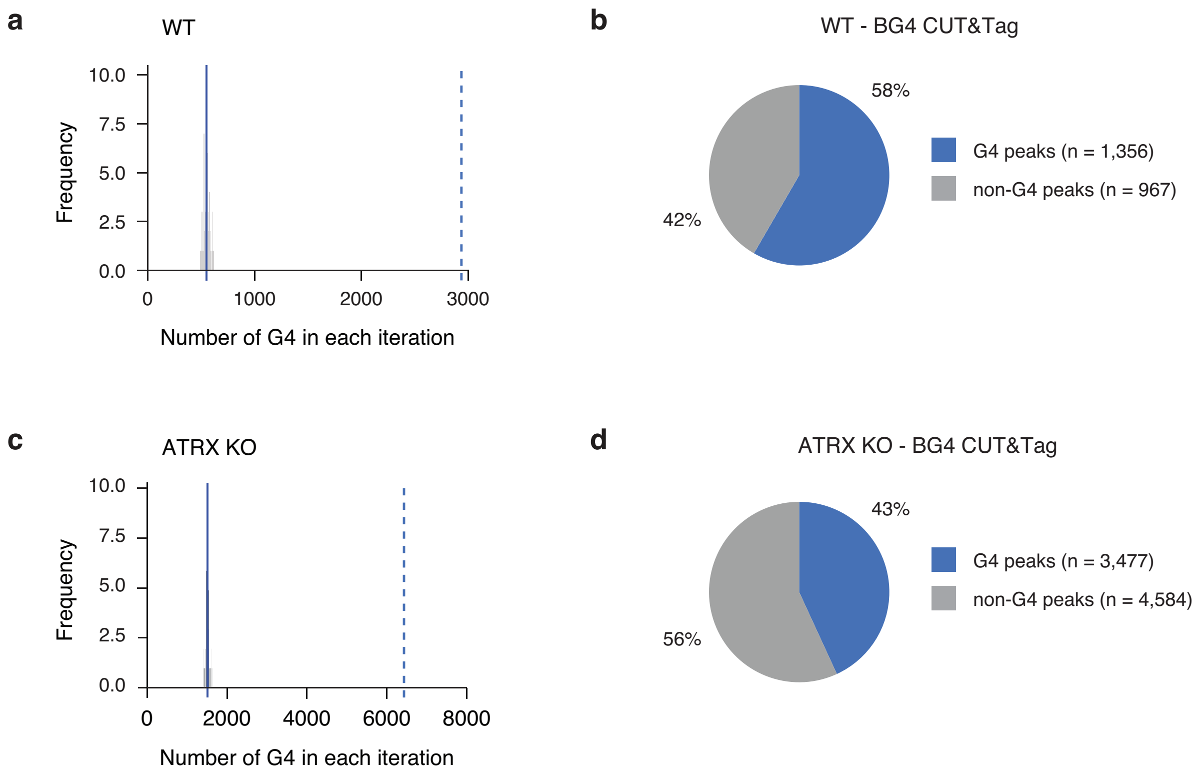

Supplementary Figure 8. G4 prediction on observed G4 peaks of CUT&Tag. Related to Figure 3. (**a**) The histogram shows the number of G4 motifs in random sequences compared with the 2,936 observed number of G4 motifs (dashed line) identified in WT ESCs. Simulation was used to estimate the number of G4 motifs by chance. For 100 times, the peaks were randomly shuffled throughout the genome while maintaining the number of peaks and their size and the number of G-quadruplexes computed. On average, 550 G-quadruplexes (sd= 28) (solid line) were observed therefore the observed number of G-quadruplex motifs is significantly larger (by 85 standard deviations) than expected by chance. (**b**) BG4 CUT&Tag analysis in WT ESCs. Pie chart represents the percentage of BG4-enriched regions containing G4 consensus motifs (1,356/2,323, 58%). (**c**) As described in panel a, but for ATRX KO ESCs. 6,428 G4 motifs were observed compared to simulation average of 1,511 (sd=45). Observed G4 motifs in ATRX KO ESCs were 110 standard deviations larger than expected by chance. (**d**) BG4 CUT&Tag analysis in ATRX KO ESCs. Pie chart represents the percentage of BG4-enriched regions containing G4 consensus motifs (3,477/8,061, 43%).

Teng et al., Supplementary Figure 9

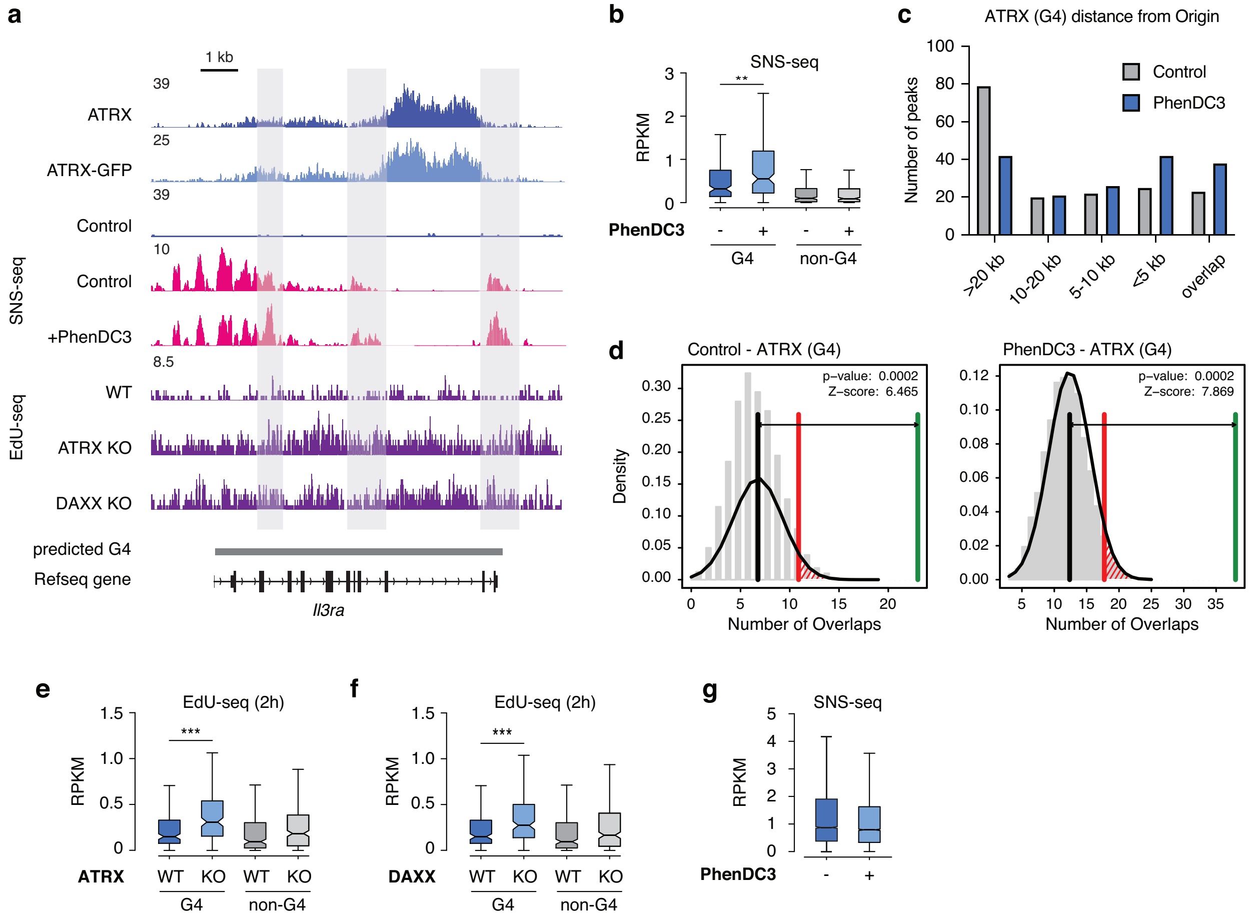

Supplementary Figure 9. Analysis of G4-related DNA synthesis and origin activity. Related to Figure 3. (**a**) Genome browser representations of ATRX, ATRX-GFP ChIP-seq, SNS-seq, and EdU-seq at predicted G4 regions in ESCs. The SNS-seq in ESCs treated with DMSO (control) or G4 stabilizer PhenDC3 (10 µM) for 48h was described previously (Prorok, P. et al. 2019). Data represented as read density in reads per kilobase per million mapped reads (RPKM) normalized to an external standard for each data set. Gray boxes indicate predicted G4 regions. (**b**) Box plots representing SNS-seq (Prorok, P. et al. 2019) in ESCs showing origin enrichment at ATRX-enriched G4 ($p = 0.008$) and non-G4 regions in the presence and absence of PhenDC3. (**c**) The distance between ATRX G4 peaks and origins defined in either SNS control or SNS PhenDC3 were determined using bedtools closest. In case of a tie, the first value is reported. The peaks were classified based on the distance to the origin in the following groups: overlap, less than 5kb, 5-10kb, 10-20kb, and more than 20 kb. The x-axis represents the distance category, and the y-axis represents the number of peaks in the particular distance category. (**d**) Statistical analysis of origin overlap shown in panel c. The number of overlaps (origins and ATRX sites) in random sequences compared with the observed number of overlaps (green line) identified in ESCs either control or PhenDC3. Simulation was used to estimate the number of overlaps by chance. For 100 times, the peaks were randomly shuffled throughout the genome while maintaining the number of peaks and their size and the number of origins computed. After simulation, the number of

overlaps (black line) were observed and the statistic was tested at $\alpha = 0.05$ (red line). (**e** and **f**) Box plots representing EdU-seq read counts at early S phase at ATRX-enriched G4 and non-G4 regions in wild-type ESCs compared to (**e**) ATRX KO ($p = 6.47 \times 10^{-5}$) and (**f**) DAXX KO ($p = 1.9 \times 10^{-4}$) ESCs. Data are representative of two independent experiments. (**g**) Box plots representing SNS-seq read counts in control or PhenDC3-treated ESCs at G4 regions experimentally identified after ATRX KO ($p = 5.9 \times 10^{-10}$). The bottom and the top of the boxes correspond to the 25th and 75th percentiles, and the internal band is the 50th percentile (median). The plot whiskers correspond to 1.5 interquartile range. Statistical significance determined by Wilcoxon Mann Whitney test. $^{**}p < 0.01$; $^{***}p < 0.001$.

Teng et al., Supplementary Fig. 10

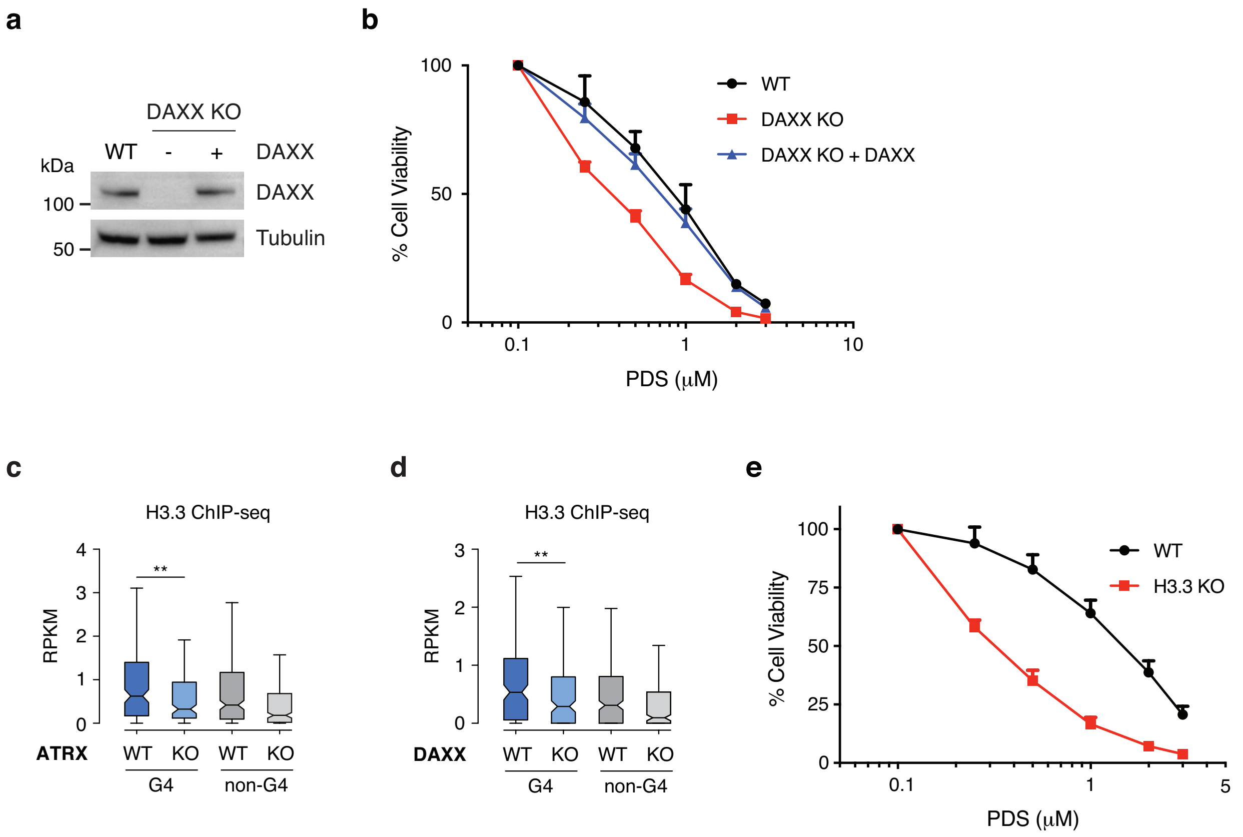

Supplementary Figure 10. DAXX KO and H3.3 KO cells are sensitive to the G4 stabilizer, PDS. Related to Figure 4. (**a**) Immunoblot from ESC whole-cell lysates showing the expression of DAXX in wild-type ESCs and DAXX KO ESCs with or without exogenous DAXX expression constructs. n=1 experimental replicate. Tubulin as loading control. (**b**) Cell viability of wild-type, DAXX KO, and DAXX KO ESCs expressing exogenous DAXX treated with PDS for 5 days. Data is one independent experiment with three technical replicates. (**c** and **d**) Box plots representing H3.3 ChIP-seq in ESCs[4] at ATRX-enriched G4 and non-G4 regions in wild-type ESCs compared to (**c**) ATRX KO ($p = 0.002$) and (**d**) DAXX KO ($p = 0.003$) ESCs. The bottom and the top of the boxes correspond to the 25th and 75th percentiles, and the internal band is the 50th percentile (median). The plot whiskers correspond to 1.5 interquartile range. Statistical significance is determined by Wilcoxon Mann Whitney test. $^{**}p < 0.01$. (**e**) Cell viability of wild-type and H3.3 KO ESCs treated with PDS for 5 days. Data are representative of two independent experiments. For **b** and **e**, mock-treated cells at day 0 were taken as 100% survival. Data represented as mean ± SD. For all immunoblots, source data are provided as a Source Data file.

Teng et al., Supplementary Fig. 11

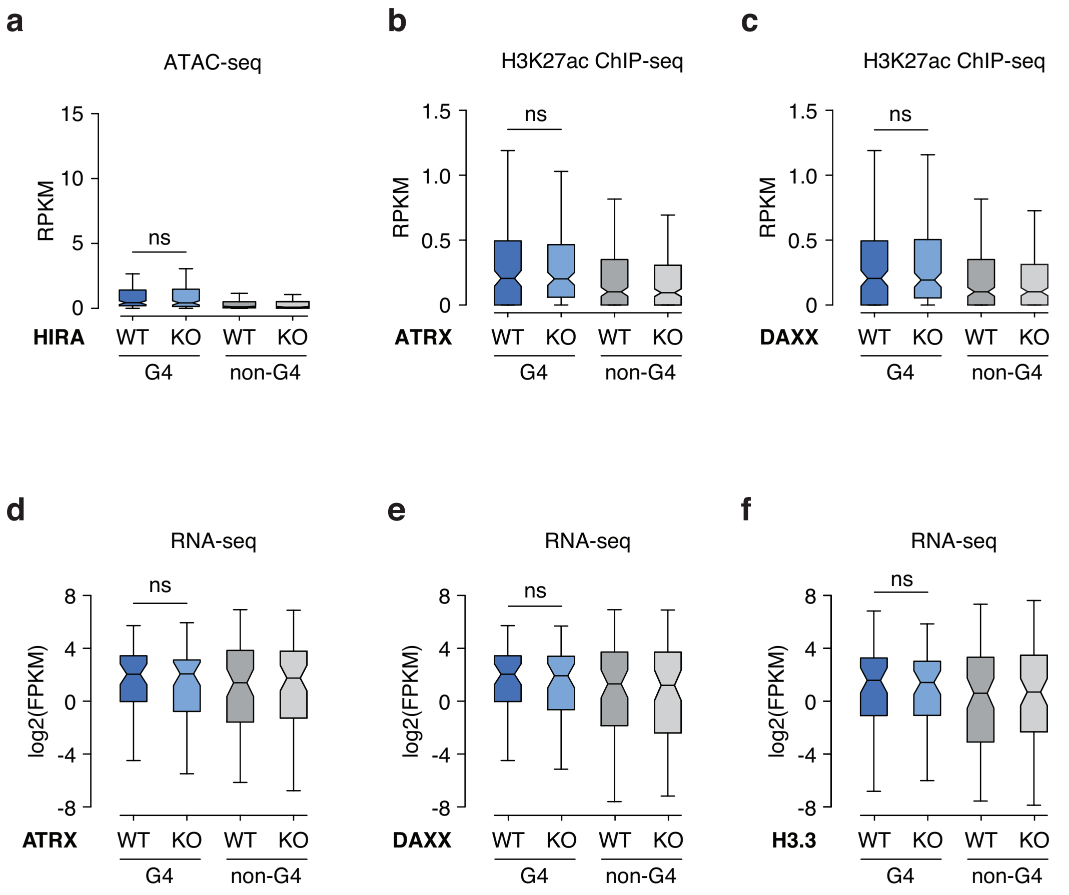

Supplementary Figure 11. ATRX/DAXX complex does not contribute uniformly to transcriptional activity of genes containing ATRX-enriched G4 regions. Related to Figure 5. (**a**) Box plots representing ATAC-seq read counts at ATRX-enriched G4 and non-G4 regions in wild-type ESCs compared to HIRA KO ESCs. (**b** and **c**) Box plots representing ChIP-seq read counts for H3K27ac[9] at ATRX-enriched G4 and non-G4 regions in wild-type ESCs compared to (**b**) ATRX KO and (**c**) DAXX KO cells. (**d**, **e**, and **f**) Box plots representing RNA-seq demonstrate that expression from ATRX-enriched G4 and non-G4 genes does not change in (**d**) ATRX KO (n=58), (**e**) DAXX KO (n=56), and (**f**) H3.3 KO ESCs (n=62), compared to wild-type ESCs[9]. Genic ATRX peak defined as within -3kb to +15kb from promoter (n=69). The bottom and the top of the boxes correspond to the 25th and 75th percentiles, and the internal band is the 50th percentile (median). The plot whiskers correspond to 1.5 interquartile range. Statistical significance is determined by Wilcoxon Mann Whitney test. ns, not significant.

Teng et al., Supplementary Fig. 12

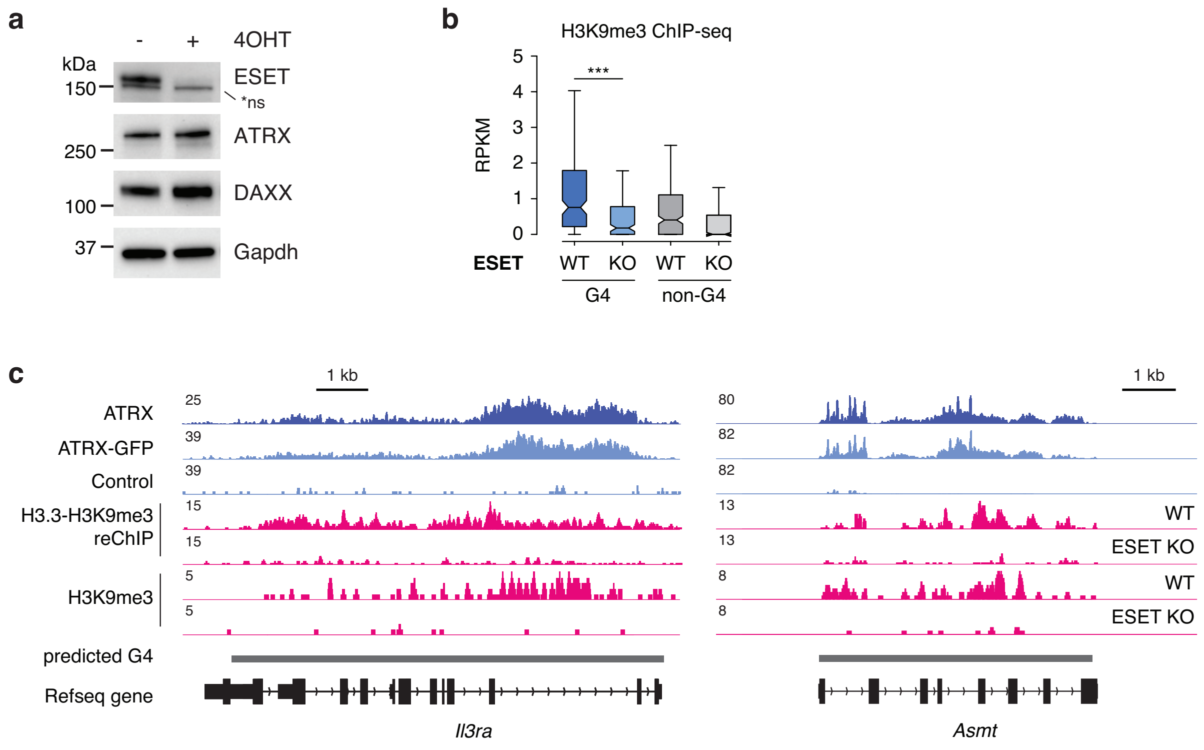

Supplementary Figure 12. ESET-mediated heterochromatin formation at ATRX-enriched G4 regions. Related to Figure 7. (**a**) Immunoblot from ESC whole-cell lysates showing the expression of ESET, ATRX, and DAXX in wild-type and ESET KO (+ 4OHT) ESCs, n>3. Gapdh as loading control. (**b**) Box plots representing ChIP-seq read counts for H3K9me3[10] at ATRX-enriched G4 ($p = 7.21 \times 10^{-9}$) and non-G4 regions in wild-type ESCs compared to ESET KO cells. The bottom and the top of the boxes correspond to the 25th and 75th percentiles, and the internal band is the 50th percentile (median). The plot whiskers correspond to 1.5 interquartile range. Statistical significance is determined by Wilcoxon Mann Whitney test. ***$p < 0.001$. (**c**) Genome browser representations of ATRX, ATRX-GFP, H3K9me3 ChIP-seq[10] and H3.3-H3K9me3 reChIP-seq[4] at predicted G4 regions in ESCs. For all immunoblots, source data are provided as a Source Data file.

Teng et al., Supplementary Figure 13

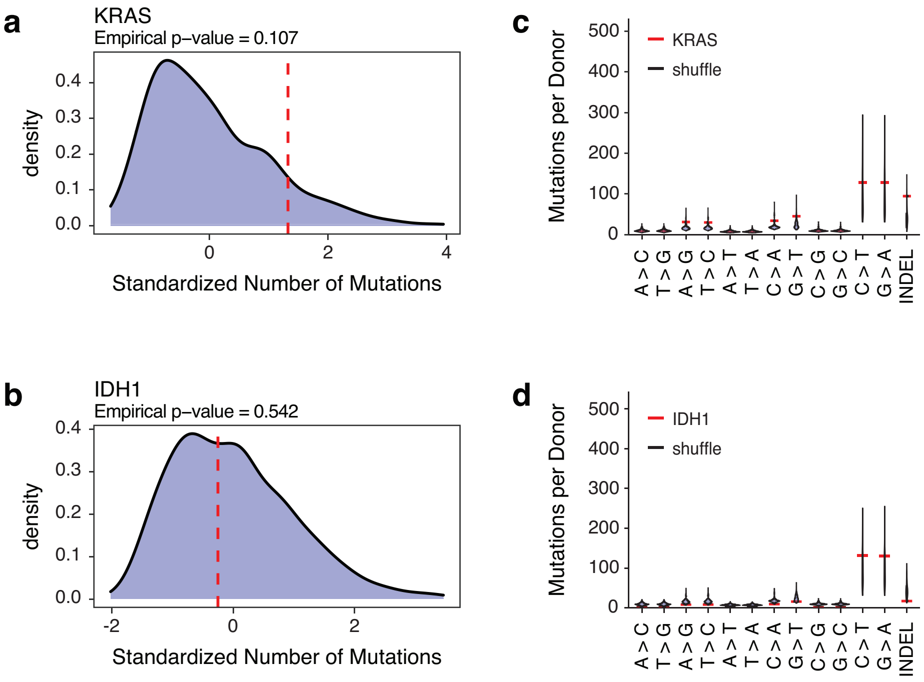

Supplementary Figure 13. Mutations at G4 regions are not correlated with *KRAS* or *IDH1* mutations in human tumors. Related to Figure 8. (**a** and **b**) Red line shows standardized number of mutations at G4 regions in *KRAS* (n = 364) (**a**) or *IDH1* (n = 571) (**b**) mutant tumors. Histograms show the mutation density at observed G4 regions in an iteratively (n = 504) and randomly selected patient cohort of the same size. (**c** and **d**) Analysis of single-nucleotide mutations and insertion-deletion (INDEL) mutations in the *KRAS* (**c**) or *IDH1* (**d**) mutant tumors compared with the shuffled patient cohort described above. Mutations per donor from the shuffled cohort are displayed as a violin plot in which the width of the shaded area represents the proportion of data located there.

Supplementary Table 1 List of published genomic data sets analyzed in this study

Experiment	Used in Figure #	SampleName	GSM # or SRX #	Published GEO filename or library name of SRX #
XChIP-seq	Supplemental Figure 1d, 1k	ATRX_ChIP	GSM551138	ATRX_Mouse_ES_ChIPseq
XChIP-seq	Figure 7c	ESC_ESETWT_H33	GSM1555153	ESC_ESETWT_H33 (ctrl for ESET KO)
XChIP-seq	Figure 7c	ESC_ESETKO_H33	GSM1555154	ESC_ESETKO_H33
XChIP-seq	Supplemental Figure 10c	ATRXWT-H33BHA_HA_ChIP	GSM1555141	ESC_ATRXWT-H33BHA_HA (ctrl for ATRX KO)
XChIP-seq	Supplemental Figure 10c	ATRXnull-H33BHA_HA_ChIP	GSM1555142	ESC_ATRXnull-H33BHA_HA
NChIP-seq	Supplemental Figure 10d	DAXXWT_H33-HA_ChIP	GSM1429928	ESC_DAXXWT_H33-HA (ctrl for DAXXnull)
NChIP-seq	Supplemental Figure 10d	DAXXnull_H33-HA_ChIP	GSM1429929	ESC_DAXXnull_H33-HA
NChIP-seq	Figure 6b	ATRXWT_H3K9me3_ChIP	GSM1555143	ESC_ATRXWT_H3K9me3 (ctrl for ATRX KO)
NChIP-seq	Figure 6b	ATRXnull_H3K9me3_ChIP	GSM1555144	ESC_ATRXnull_H3K9me3
NChIP-seq	Figure 6c	DAXXWT_H3K9me3_ChIP	GSM1555134	ESC_DAXXWT_H3K9me3 (ctrl for DAXX KO)
NChIP-seq	Figure 6c	DAXXnull_H3K9me3_ChIP	GSM1555135	ESC_DAXXnull_H3K9me3
NChIP-seq	Figure 6d	H33WT_H3K9me3_ChIP	GSM1555131	ESC_H33WT_nH3K9me3 (ctrl for KO)
NChIP-seq	Figure 6d	H33KO1_H3K9me3_ChIP	GSM1555132	ESC_H33KO1_nH3K9me3
NChIP-seq	Figure 7b	ESETWT_H33-H3K9me3_ChIP	GSM1555155	ESC_ESETWT_H33-H3K9me3 (ctrl for ESET KO)
NChIP-seq	Figure 7b	ESETKO_H33-H3K9me3_ChIP	GSM1555156	ESC_ESETKO_H33-H3K9me3
NChIP-seq	Supplemental Figure 12b	H3K9me3-SL-ChIP-seq-no4HT	SRX5891228	H3K9me3 ChIP-seq of Setdb1-CKO mESC in SL condition (no 4OHT)
NChIP-seq	Supplemental Figure 12b	H3K9me3-SL-ChIP-seq-4HT	SRX5891224	H3K9me3 ChIP-seq of Setdb1-CKO mESC in SL condition (add 4OHT)
NChIP-seq	Supplemental Figure 11b, 11c	ATRXDAXXWT_H3K27ac_ChIP	GSM3143877	ESC_ATRXDAXXWT_H3K27ac (ctrl for ATRX & DAXX KO)
NChIP-seq	Supplemental Figure 11b	ATRXKO_H3K27ac_ChIP	GSM3143878	ESC_ATRXKO_H3K27ac
NChIP-seq	Supplemental Figure 11c	DAXXKO_H3K27ac_ChIP	GSM3143879	ESC_DAXXKO_H3K27ac
ATAC-seq	Figure 5d	H33WT_ATAC	GSM3143855	ESC_H33WT_ATAC (ctl for H3.3 KO)
ATAC-seq	Figure 5d	H33KO_ATAC	GSM3143856	ESC_H33KO_ATAC
ATAC-seq	Figure 5f	ESC H3.3KO	GSM4490207	ESC H3.3KO
ATAC-seq	Figure 5f	ESC H3.3KO +H3.3	GSM4490208	ESC H3.3KO +H3.3
ATAC-seq	Figure 5f	ESC H3.3KO +H3.2	GSM4490209	ESC H3.3KO +H3.2
ATAC-seq	Figure 5f	ESC H3.3KO +H3.3LI->AA	GSM4490210	ESC H3.3KO +H3.3LI->AA
ATAC-seq	Supplemental Figure 11a	HIRAWT_ATAC	GSM3143857	ESC_HIRAWT_ATAC (ctl for HIRA KO1)
ATAC-seq	Supplemental Figure 11a	HIRAKO1_ATAC	GSM3143858	ESC_HIRAKO1_ATAC
RNA-seq	Supplemental Figure 11d, 11e	Wildtype_RNA-seq	GSM1905019	Wildtype
RNA-seq	Supplemental Figure 11d	ATRX KO_RNA-seq	GSM1905021	ATRX KO
RNA-seq	Supplemental Figure 11e	DAXX KO_RNA-seq	GSM1905020	DAXX KO
RNA-seq	Supplemental Figure 11f	H33WT_RNA-seq_rep1	GSM3143909	ESC_H33WT_RNA-seq_rep1 (ctl for H3.3 KO)
RNA-seq	Supplemental Figure 11f	H33WT_RNA-seq_rep2	GSM3143910	ESC_H33WT_RNA-seq_rep2 (ctl for H3.3 KO)
RNA-seq	Supplemental Figure 11f	H33KO_RNA-seq_rep1	GSM3143911	ESC_H33KO_RNA-seq_rep1
RNA-seq	Supplemental Figure 11f	H33KO_RNA-seq_rep2	GSM3143912	ESC_H33KO_RNA-seq_rep2
SNS-seq	Supplemental Figure 9b-d and 9g	SNS-seq Ctrl rep1	GSM3602315	SNS-seq Ctrl rep1
SNS-seq	Supplemental Figure 9b-d and 9g	SNS-seq Ctrl rep2	GSM3602316	SNS-seq Ctrl rep2
SNS-seq	Supplemental Figure 9b-d and 9g	SNS-seq Ctrl rep3	GSM3602317	SNS-seq Ctrl rep3
SNS-seq	Supplemental Figure 9b-d and 9g	SNS-seq PhenDC3 rep1	GSM3602318	SNS-seq PhenDC3 rep1
SNS-seq	Supplemental Figure 9b-d and 9g	SNS-seq PhenDC3 rep2	GSM3602319	SNS-seq PhenDC3 rep2
G4-seq	Figure 8, Supplemental Figure 13	Homo_Li_K	GSM3003539	Homo_Li_K
G4-seq	Figure 8, Supplemental Figure 13	Homo_Li_KPDS	GSM3003540	Homo_Li_KPDS
Tumor mutation	Figure 8, Supplemental Figure 13		Pan-cancer analysis of whole genomes (dcc.icgc.org/pcawg)	

Supplementary Table 2 List of antibodies used in this study

Target	Merchant	Catalog number	Application
ATRX	Santa Cruz	sc-55584	Immunoblot (1:500), PLA (1:100)
ATRX	Abcam	ab97508	Immunoblot (1:1000), Co-immunoprecipitation, ChIP, PLA (1:2500)
DAXX	Santa Cruz	sc-8043	Immunoblot (1:500)
DAXX	Cell Signaling	4533	Immunoblot (1:1000)
HIRA		WC15 and WC119	Immunoblot (1:1000), Co-immunoprecipitation, PLA (1:1000)
H3.3	Millipore	09-838	Immunoblot (1:500)
GFP	Abcam	ab290	Immunoblot (1:1000), Co-immunoprecipitation, ChIP
HA	Biolegend	901501	Immunoblot (1:500)
HA	Abcam	ab9110	Co-immunoprecipitation
β-Tubulin	Sigma	T5201	Immunoblot (1:10000)
Gapdh	Cell Signaling	2118	Immunoblot (1:10000)
Mcm2	Santa Cruz	sc-373702	Immunoblot (1:500)
Mcm6	Santa Cruz	sc-393618	Immunoblot (1:500)
Mcm7	Santa Cruz	sc-65469	Immunoblot (1:500)
ESET	Santa Cruz	sc-66884	Immunoblot (1:500)
anti-mouse IgG-HRP	GE	NA93V	Immunoblot
anti-rabbit IgG-HRP	Bio-Rad	170-6516	Immunoblot
normal mouse IgG	Santa Cruz	sc-2025	Co-immunoprecipitation
rabbit IgG	Jackson ImmunoResearch	011-000-003	Co-immunoprecipitation
H3K9me3	Abcam	ab8898	ChIP
Spike-in antibody	Active Motif	61686	ChIP
DNA G-quadruplex	Millipore	Clone BG4, MABE917	PLA (1:200), CUT&Tag
DYKDDDDK Tag	Cell Signaling	2368	PLA (1:1000), CUT&Tag
Biotin	Bethyl	A150-109A	PLA (1:1000)
Biotin	Jackson ImmunoResearch	200-002-211	PLA (1:1000)
Mcm2	Sigma	PLA0060	PLA (1:2500)
Mcm3	Sigma	PLA0061	PLA (1:2500)
Mcm4	Sigma	PLA0062	PLA (1:2500)
Mcm6	Sigma	PLA0041	PLA (1:2500)
RNA Pol II	Bethyl	A300-654A	PLA (1:1000)
anti-rabbit secondary antibody	EpiCypher	13-0047	CUT&Tag

Supplementary Table 3 List of primers used for ChIP H3K9me3

Chr	Start (mm10)	End (mm10)	Forward sequences (5'-> 3')	Reverse sequences (5'-> 3')
chr5	32706605	32706841	TACTATCAGGTCGGGGGAGC	CCCGGTCTAGGTTGAAGCAA
chr7	137516417	137516532	AATGCAGTCGTTTGTGGCTC	GGGGAGACATTGTAGCTCGT
chr1	182501262	182501337	AAAGCCCCACTCCCTGTTTC	GCCCAGCCTAGTTGGTGTAA
chr13	119489112	119489260	TGTAAAGCCGGATGAAGGCA	CTCAGGGAACAATCACGAGC
chrX	170675686	170675755	TGGTGCAGGAATTGACGGG	CCTCCGACCTGTCAGTCAAA
chr7	7299659	7299734	CGCTGCAGACGGGAGG	CTGGGGAAAGCGAGACAGAC
chr7	7278070	7278168	CTGTCCCTGCACTCCTACAG	AGACGAGCGGTGTGGC

References

1. Ballabeni, A. *et al.* Cell cycle adaptations of embryonic stem cells. *Proc. Natl. Acad. Sci. U. S. A.* **108**, 19252–19257 (2011).
2. Law, M. J. *et al.* ATR-X syndrome protein targets tandem repeats and influences allele-specific expression in a size-dependent manner. *Cell* **143**, 367–378 (2010).
3. Truch, J., Telenius, J., Higgs, D. R. & Gibbons, R. J. How to Tackle Challenging ChIP-Seq, with Long-Range Cross-Linking, Using ATRX as an Example. *Methods Mol. Biol.* **1832**, 105–130 (2018).
4. Elsässer, S. J., Noh, K.-M., Diaz, N., Allis, C. D. & Banaszynski, L. A. Histone H3.3 is required for endogenous retroviral element silencing in embryonic stem cells. *Nature* **522**, 240–244 (2015).
5. Buenrostro, J. D., Giresi, P. G., Zaba, L. C., Chang, H. Y. & Greenleaf, W. J. Transposition of native chromatin for fast and sensitive epigenomic profiling of open chromatin, DNA-binding proteins and nucleosome position. *Nat. Methods* **10**, 1213–1218 (2013).
6. Kaya-Okur, H. S. *et al.* CUT&Tag for efficient epigenomic profiling of small samples and single cells. *Nat. Commun.* **10**, 1930 (2019).
7. Lyu, J., Shao, R. & Elsässer, S. J. Genome-wide mapping of G-quadruplex structures with CUT&Tag. *bioRxiv* (2021).
8. Macheret, M. & Halazonetis, T. D. Monitoring early S-phase origin firing and replication fork movement by sequencing nascent DNA from synchronized cells. *Nat. Protoc.* **14**, 51–67 (2019).
9. Martire, S. *et al.* Phosphorylation of histone H3.3 at serine 31 promotes p300 activity and enhancer acetylation. *Nat. Genet.* **51**, 941–946 (2019).
10. Wu, K. *et al.* SETDB1-Mediated Cell Fate Transition between 2C-Like and Pluripotent States. *Cell Rep.* **30**, 25–36.e6 (2020).
