# Supplementary material for: ATRX promotes heterochromatin formation to protect cells from G-quadruplex DNA-mediated stress
Source: Nat Commun. 2021 Jun 23;12:3887. doi: 10.1038/s41467-021-24206-5 (PMC8222256; doi:10.1038/s41467-021-24206-5)
Supplement: Supplementary file 3 — Description of Additional Supplementary Files [file 41467_2021_24206_MOESM3_ESM.pdf]

Description of Additional Supplementary Files

File Name: Supplementary Data 1
Description: List of ATRX-bound G4 and ATRX-bound non-G4 regions

File Name: Supplementary Data 2
Description: List of ATRX-interacting proteins in HeLa WT and ATRX KO cells
