# Supplementary material for: ATRX promotes heterochromatin formation to protect cells from G-quadruplex DNA-mediated stress
Source: Nat Commun. 2021 Jun 23;12:3887. doi: 10.1038/s41467-021-24206-5 (PMC8222256; doi:10.1038/s41467-021-24206-5)
Supplement: Supplementary file 6 — Reporting Summary [file 41467_2021_24206_MOESM6_ESM.pdf]

nature research

Corresponding author(s): Laura Banaszynski

Last updated by author(s): May 18, 2021

# Reporting Summary

Nature Research wishes to improve the reproducibility of the work that we publish. This form provides structure for consistency and transparency in reporting. For further information on Nature Research policies, see our Editorial Policies and the Editorial Policy Checklist.

## Statistics

For all statistical analyses, confirm that the following items are present in the figure legend, table legend, main text, or Methods section.

| n/a | Confirmed | |
|---|---|---|
| ☐ | ☒ | The exact sample size (*n*) for each experimental group/condition, given as a discrete number and unit of measurement |
| ☐ | ☒ | A statement on whether measurements were taken from distinct samples or whether the same sample was measured repeatedly |
| ☐ | ☒ | The statistical test(s) used AND whether they are one- or two-sided<br>*Only common tests should be described solely by name; describe more complex techniques in the Methods section.* |
| ☒ | ☐ | A description of all covariates tested |
| ☒ | ☐ | A description of any assumptions or corrections, such as tests of normality and adjustment for multiple comparisons |
| ☐ | ☒ | A full description of the statistical parameters including central tendency (e.g. means) or other basic estimates (e.g. regression coefficient) AND variation (e.g. standard deviation) or associated estimates of uncertainty (e.g. confidence intervals) |
| ☐ | ☒ | For null hypothesis testing, the test statistic (e.g. *F*, *t*, *r*) with confidence intervals, effect sizes, degrees of freedom and *P* value noted<br>*Give P values as exact values whenever suitable.* |
| ☒ | ☐ | For Bayesian analysis, information on the choice of priors and Markov chain Monte Carlo settings |
| ☒ | ☐ | For hierarchical and complex designs, identification of the appropriate level for tests and full reporting of outcomes |
| ☒ | ☐ | Estimates of effect sizes (e.g. Cohen's *d*, Pearson's *r*), indicating how they were calculated |

*Our web collection on statistics for biologists contains articles on many of the points above.*

## Software and code

Policy information about availability of computer code

| | |
|---|---|
| Data collection | BD FACSDiva Software (v. 8.0.2)<br>BD FACSCanto II System Software (v. 6.0)<br>ChemiDoc MP Imaging System (v. 2.3.0.07)<br>softWoRx (v. 7.0.0)<br>LightCycler® 480 System Performance Data Software (v. 1.5.1)<br>BMG Labtech Control Software (v. 5.21 R2)<br>MARS Data Analysis Software (v. 3.20 R2) |
| Data analysis | FastQC (v. 0.10.1), BOWTIE2 (v. 2.3.2), BWA (v. 0.7.5), SAMtools (v. 1.6), BEDtools (2.25.0), Integrative Genomics Viewer (IGV v. 2.3), UCSC genome browser, MACS (v. 2.1.2), TopHat (v. 2.0.9), Cufflinks (v. 2.2.1), Cuffdiff, R (v. 3.2.1), Prism (v. 8), Excel (v. 16.18), Deeptools (v. 3.0.2), FlowJo (v. 10.3.0), Image Lab (v. 6.0.0), ImageJ (v. 1.49u)<br><br>https://github.com/utsw-medical-center-banaszynski-lab/Teng-et-al-2021-Nature-Communications |

For manuscripts utilizing custom algorithms or software that are central to the research but not yet described in published literature, software must be made available to editors and reviewers. We strongly encourage code deposition in a community repository (e.g. GitHub). See the Nature Research guidelines for submitting code & software for further information.

# Data

Policy information about availability of data

All manuscripts must include a data availability statement. This statement should provide the following information, where applicable:

- Accession codes, unique identifiers, or web links for publicly available datasets
- A list of figures that have associated raw data
- A description of any restrictions on data availability

Datasets are generated during the current study are deposited in the NCBI Gene Expression Omnibus using series accession number GSE151058 (https://www.ncbi.nlm.nih.gov/geo/query/acc.cgi?acc=GSE151058). Subseries are GSE151053 (ATAC-seq), GSE151054 (ChIP-seq), GSE151056 (EdU-seq), GSE171461 (CUT&Tag). The mass spectrometry proteomics data have been deposited to MassIVE with the dataset identifier MSV000085543. Source data are provided with this paper.

# Field-specific reporting

Please select the one below that is the best fit for your research. If you are not sure, read the appropriate sections before making your selection.

☒ Life sciences ☐ Behavioural & social sciences ☐ Ecological, evolutionary & environmental sciences

For a reference copy of the document with all sections, see nature.com/documents/nr-reporting-summary-flat.pdf

# Life sciences study design

All studies must disclose on these points even when the disclosure is negative.

| | |
|---|---|
| Sample size | ATAC-seq studies were performed in duplicate. The majority of the ChiP-Seq experiments were performed at least in duplicate. Imaging figures were generated with a variety of replicates as reported in the figure legends of our manuscript. Sample sizes were chosen based on literature precedent. |
| Data exclusions | No data were excluded from the analysis. |
| Replication | Critical ChIP-seq and ATAC-seq data sets were obtained in duplicate. Many ChIP-seq data sets were validated with independent ChIP-qPCR assays, with orthogonal ChIP approaches, or using KO cells. All cell viability assays, immunoprecipitations, western blots and related experiments were performed in duplicate or more. Results obtained among replicates were consistent. |
| Randomization | Random allocation is not relevant to our study for most assays, since we are performing molecular and genomic analysis of known KO mESCs. We performed randomization of patient samples as controls for our analysis of patient mutation data, as described in the methods. |
| Blinding | Blinding was not relevant to our study; our techniques were all molecular biological techniques where the experimenter designs and executes the experimental conditions so blinding is not possible. |

# Reporting for specific materials, systems and methods

We require information from authors about some types of materials, experimental systems and methods used in many studies. Here, indicate whether each material, system or method listed is relevant to your study. If you are not sure if a list item applies to your research, read the appropriate section before selecting a response.

## Materials & experimental systems

| n/a | Involved in the study | |
|---|---|---|
| ☐ | ☒ | Antibodies |
| ☐ | ☒ | Eukaryotic cell lines |
| ☒ | ☐ | Palaeontology and archaeology |
| ☒ | ☐ | Animals and other organisms |
| ☒ | ☐ | Human research participants |
| ☒ | ☐ | Clinical data |
| ☒ | ☐ | Dual use research of concern |

## Methods

| n/a | Involved in the study | |
|---|---|---|
| ☐ | ☒ | ChIP-seq |
| ☐ | ☒ | Flow cytometry |
| ☒ | ☐ | MRI-based neuroimaging |

## Antibodies

| | |
|---|---|
| Antibodies used | Antibodies. For chromatin immunoprecipitation, GFP (ab290, Abcam), ATRX (ab97508, Abcam), H3K9me3 (ab8898, Abcam), and Spike-in antibody (61686, Active Motif). For co-immunoprecipitation, GFP (ab290, Abcam), HA (ab9110, Abcam), ATRX (ab97508, Abcam), HIRA (mouse monoclonal WC15 and WC119), normal mouse IgG (sc-2025, Santa Cruz) and rabbit IgG (011-000-003, Jackson ImmunoResearch). For immunoblot, ATRX (sc-55584, Santa Cruz and ab97508, Abcam), DAXX (sc-8043, Santa Cruz; 4533, Cell Signaling), HIRA (mouse monoclonal WC15 and WC119), H3.3 (09-838, Millipore), GFP (ab290, Abcam), HA (901501, Biolegend), β- |

| | |
|---|---|
| | Tubulin (T5201, Sigma), Gapdh (2118, Cell Signaling), Mcm2 (sc-373702, Santa Cruz), Mcm6 (sc-393618, Santa Cruz), Mcm7 (sc-65469, Santa Cruz), ESET (sc-66884, Santa-Cruz), anti-mouse IgG-HRP (NA93V, GE), and anti-rabbit IgG-HRP (170-6516, Bio-Rad). For proximity ligation assay, ATRX (sc-55584, Santa Cruz and ab97508, Abcam), DNA G-quadruplex (Clone BG4, MABE917, Millipore), DYKDDDDK Tag (2368, Cell Signaling), Biotin (A150-109A, Bethyl and 200-002-211, Jackson ImmunoResearch), Mcm2 (PLA0060, Sigma), Mcm3 (PLA0061, Sigma), Mcm4 (PLA0062, Sigma), Mcm6 (PLA0041, Sigma), HIRA (WC15 and WC119), RNA Pol II (A300-654A, Bethyl). For Cleavage Under Targets & Tagmentation (CUT&Tag), DNA G-quadruplex (Clone BG4, MABE917, Millipore), DYKDDDDK Tag (2368, Cell Signaling), and anti-rabbit secondary antibody (13-0047, EpiCypher). |
| Validation | Many histone modification antibodies, including H3K9me3 used in our study, have been validated using Brian Strahl's histone tail peptide array (http://histoneantibodies.com). We validated the H3.3, ESET, HIRA, ATRX, and DAXX antibodies using our KO mESCs, where no signal was observed for each antibody in its corresponding KO cell line. GFP antibodies were validated based on lack of signal in cell lines not expressing GFP. Likewise for HA and Flag antibodies. |

# Eukaryotic cell lines

Policy information about cell lines

| | |
|---|---|
| Cell line source(s) | H3.3 WT and KO mESCs - Banaszynski et al., Cell 2013; WT and ATRX KO and DAXX KO mESCs- Sadic et al., EMBO Rep 2015; HIRA KO - Martire et al. Nature Genetics 2019; ESET cKO - Matsui et al. Nature 2010; HeLa and 293T from ATCC |
| Authentication | KO cell lines were validated by sequencing of genomic DNA and by immunoblot of the protein product. WT cell lines are routinely karotyped to confirm ploidy. HeLa and 293T were purchased directly from ATCC. |
| Mycoplasma contamination | All cell lines tested were negative for mycoplasma. Cells were tested monthly. |
| Commonly misidentified lines (See ICLAC register) | None |

# ChIP-seq

## Data deposition

☒ Confirm that both raw and final processed data have been deposited in a public database such as GEO.

☒ Confirm that you have deposited or provided access to graph files (e.g. BED files) for the called peaks.

| | |
|---|---|
| Data access links *May remain private before publication.* | To review GEO accession GSE151058:<br>Go to https://www.ncbi.nlm.nih.gov/geo/query/acc.cgi?acc=GSE151058 |
| Files in database submission | Edu-seq<br>ESC_WT_EdU2h_rep1<br>ESC_ATRXKO_EdU2h_rep1<br>ESC_DAXXKO_EdU2h_rep1<br>ESC_WT_EdU2h_rep2<br>ESC_ATRXKO_EdU2h_rep2<br>ESC_DAXXKO_EdU2h_rep2<br>ESC_WT_Input2h_rep1<br><br>ATAC-seq<br>ESC_WT_ATAC_rep1<br>ESC_ATRXKO_ATAC_rep1<br>ESC_DAXXKO_ATAC_rep1<br>ESC_WT_ATAC_rep2<br>ESC_ATRXKO_ATAC_rep2<br>ESC_DAXXKO_ATAC_rep2<br>ESC_HIRAKO_ATAC_rep2<br>ESC_ATRXKO-Ctl_ATAC_rep1<br>ESC_ATRXKO-WT_ATAC_rep1<br>ESC_ATRXKO-L1238A_ATAC_rep1<br>ESC_ATRXKO-K1562R_ATAC_rep1<br>ESC_ATRXKO-Ctl_ATAC_rep2<br>ESC_ATRXKO-WT_ATAC_rep2<br>ESC_ATRXKO-L1238A_ATAC_rep2<br>ESC_ATRXKO-K1562R_ATAC_rep2<br>ESC_ESETcKO_DMSO_ATAC_rep1<br>ESC_ESETcKO_4OHT_ATAC_rep1<br>ESC_ESETcKO_DMSO_ATAC_rep2<br>ESC_ESETcKO_4OHT_ATAC_rep2<br><br>ChIP-seq<br>ESC_ATRXWT_ATRX |

ESC_ATRXKO_ATRX
ESC_ATRX-GFP_GFP
ESC_ATRXWT_GFP

CUT&Tag
ESC_ATRXWT_BG4
ESC_ATRXKO_BG4

Genome browser session (e.g. UCSC)

No longer applicable.

## Methodology

Replicates

All EdU-seq and ATAC-seq experiments were performed in duplicate.

Sequencing depth

Sample / Total Reads / Unique Reads / Read length / PE or SE
EdU-seq
ESC_WT_EdU2h_rep1 70,837,331 46,409,661 75 bp PE
ESC_ATRXKO_EdU2h_rep1 154,331,244 43,429,332 75 bp PE
ESC_DAXXKO_EdU2h_rep1 161,681,964 95,445,753 75 bp PE
ESC_WT_EdU2h_rep2 66,189,566 47,219,434 75 bp PE
ESC_ATRXKO_EdU2h_rep2 48,938,091 33,432,013 75 bp PE
ESC_DAXXKO_EdU2h_rep2 52,334,184 35,551,484 75 bp PE
ESC_WT_Input2h_rep1 39,295,717 24,606,938 75 bp PE

ATAC-seq
ESC_WT_ATAC_rep1 5,250,227 2,177,820 75 bp PE
ESC_ATRXKO_ATAC_rep1 6,253,762 2,552,777 75 bp PE
ESC_DAXXKO_ATAC_rep1 4,752,582 1,952,910 75 bp PE
ESC_WT_ATAC_rep2 31,874,761 18,670,767 75 bp PE
ESC_ATRXKO_ATAC_rep2 48,141,647 26,740,358 75 bp PE
ESC_DAXXKO_ATAC_rep2 44,885,481 28,141,213 75 bp PE
ESC_HIRAKO_ATAC_rep2 46,596,788 26,753,748 75 bp PE
ESC_ATRXKO-Ctl_ATAC_rep1 34,937,967 20,838,482 75 bp PE
ESC_ATRXKO-WT_ATAC_rep1 29,972,493 19,125,144 75 bp PE
ESC_ATRXKO-L1238A_ATAC_rep1 33,793,021 21,469,873 75 bp PE
ESC_ATRXKO-K1562R_ATAC_rep1 42,423,228 26,239,934 75 bp PE
ESC_ATRXKO-Ctl_ATAC_rep2 38,252,288 22,378,938 75 bp PE
ESC_ATRXKO-WT_ATAC_rep2 22,316,283 14,170,375 75 bp PE
ESC_ATRXKO-L1238A_ATAC_rep2 32,749,560 20,424,563 75 bp PE
ESC_ATRXKO-K1562R_ATAC_rep2 27,192,198 16,773,419 75 bp PE
ESC_ESETcKO_DMSO_ATAC_rep1 40,806,510 20,519,286 75 bp PE
ESC_ESETcKO_4OHT_ATAC_rep1 28,829,869 14,889,150 75 bp PE
ESC_ESETcKO_DMSO_ATAC_rep2 29,716,783 15,677,019 75 bp PE
ESC_ESETcKO_4OHT_ATAC_rep2 35,002,492 17,022,056 75 bp PE

ChIP-seq
ESC_ATRXWT_ATRX 28,525,913 12,693,834 75 bp PE
ESC_ATRXKO_ATRX 26,086,886 12,626,824 75 bp PE
ESC_ATRX-GFP_GFP 13,615,551 6,258,591 75 bp PE
ESC_ATRXWT_GFP 16,598,897 6,003,734 75 bp PE

CUT&Tag (Optical duplicates removed)
ESC_ATRXWT_BG4 23,992,743 23,321,635 75 bp PE
ESC_ATRXKO_BG4 32,000,638 30,892,539 75 bp PE

Antibodies

For chromatin immunoprecipitation, GFP (ab290, Abcam), ATRX (ab97508, Abcam), H3K9me3 (ab8898, Abcam), and Spike-in antibody (61686, Active Motif). For Cleavage Under Targets & Tagmentation (CUT&Tag), DNA G-quadruplex (Clone BG4, MABE917, Millipore), DYKDDDDK Tag (2368, Cell Signaling), and anti-rabbit secondary antibody (13-0047, EpiCypher).

Peak calling parameters

macs2 callpeak -t $experiment -c $control -f BED -n $out_dir/$prefix -g $genomesize -p 1e-2 --mfold 10,50 --nomodel --shift 0 --extsize $fraglen --keep-dup all -B --SPMR

Data quality

FastQC tool was used to perform a quality check on all sequencing data to assess the quality of the raw reads and identify possible sequencing errors or biases to ensure that the raw data looks good and can be used for further downstream analyses. Peak calling by MACS2 reported over 70% of peaks that had above 5-fold enrichment. Enrichment metrics of pvalue 1e-2 and mfold 10,50 were used for calling peaks to ensure high-confidence enrichment against background.

Software

FastQC, BOWTIE, BWA, SAMtools, BEDtools, MACS2, R, Deeptools

# Flow Cytometry

## Plots

Confirm that:

☒ The axis labels state the marker and fluorochrome used (e.g. CD4-FITC).

☒ The axis scales are clearly visible. Include numbers along axes only for bottom left plot of group (a 'group' is an analysis of identical markers).

☒ All plots are contour plots with outliers or pseudocolor plots.

☒ A numerical value for number of cells or percentage (with statistics) is provided.

## Methodology

| | |
|---|---|
| Sample preparation | Cells were incubated in medium with 2 mM thymidine for 14h, washed, and incubated in medium with 50 ngml-1 nocodazole for 7h1. After wash, mitotic cells were labeled with 10 µM EdU for 30 min prior to fixation. 1x 106 cells were trypsinized, washed with 1% BSA/PBS, fixed with Click-it fixative, permeabilized with Click-it saponin-based permeabilization and wash reagent and incubated with Click-it reaction (see kit manual). Cells were washed and resuspended in Click-it saponin-based permeabilization and wash reagent with 2 drops of SYTOX AADvancedTM Ready Flow ReagentTM (Thermo Fisher, R37173). |
| Instrument | Flow cytometry performed on a BD FACSCantoTM II (BD Biosciences). |
| Software | Cell cycle analysis was performed using FlowJo software. |
| Cell population abundance | All cells were considered for analysis. |
| Gating strategy | SYTOX ADD was used to gate 2n and 4n cells on the x axis. EdU-488 intensity was used to gate cells that (1) were in S-phase during the labeling procedure, or (2) in either G1 or G2/M during the labeling procedure. |

☒ Tick this box to confirm that a figure exemplifying the gating strategy is provided in the Supplementary Information.
